# Immune-Checkpoint-Inhibitor-Related Cardiovascular Toxicities in Cancer: A Mechanistic Review of Molecular Pathways with AI-Assisted Literature Clustering

**DOI:** 10.3390/ijms27104378

**Published:** 2026-05-14

**Authors:** Ileana-Raluca Pătru, Dimitrie-Ionuț Atasiei, Radu Tudor Ionescu, Alina Gabriela Negru, Ionut-Lucian Antone-Iordache, Maria Iordache, Alexandra Valentina Anghel, Andreea-Iuliana Ionescu

**Affiliations:** 1 Faculty of Medicine, “Carol Davila” University of Medicine and Pharmacy, 020021 Bucharest, Romania; ileana-raluca.patru@drd.umfcd.ro (I.-R.P.); antoneiordachelucian@stud.umfcd.ro (I.-L.A.-I.); maria.malai@drd.umfcd.ro (M.I.); alexandra-valentina.anghel@stud.umfcd.ro (A.V.A.); andreea-iuliana.ionescu@umfcd.ro (A.-I.I.); 2 Medical Oncology Department, Colțea Clinical Hospital, 030167 Bucharest, Romania; 3 Neurosurgery Department, Emergency Clinical Hospital “Sf. Pantelimon”, 021659 Bucharest, Romania; 4 Department of Computer Science, University of Bucharest, 010041 Bucharest, Romania; radu.ionescu@fmi.unibuc.ro; 5 Cardiology Department, University of Medicine and Pharmacy “Victor Babes” Timisoara, 300041 Timisoara, Romania; alinanegru@umft.ro; 6 Radiotherapy Department, Colțea Clinical Hospital, 030167 Bucharest, Romania

**Keywords:** PD-1, PD-L1, immune checkpoint inhibitors, immune-related myocarditis, mechanistic framework, cardio-oncology, artificial intelligence

## Abstract

Since the first approval of CTLA-4 blockade for melanoma, immune checkpoint inhibitors (ICIs) have expanded into a major class of cancer therapy, with more than 100 FDA-approved oncological indications across metastatic and earlier-stage disease settings, including use as monotherapy and in combination regimens. Preclinical research has largely focused on myocarditis and atherosclerosis, but a wider set of phenotypes, such as non-inflammatory left ventricular dysfunction (NILVD), arrhythmias, and vasculitis, can be observed, and they are rarely connected within a single mechanistic model. We aim to build a systems-oriented, mechanistic framework of the most widely studied biological processes; it will link the main checkpoint pathways to relevant cardiac and vascular cell types, molecular pathways, immune synapses, and candidate biomarkers. We searched PubMed, Scopus, and Web of Science using combinations of terms for immune checkpoint inhibition and cardiovascular-immune-related adverse events that provide mechanistic insight into cardiac-immune-related adverse reactions (irAEs). An AI-assisted semantic clustering approach was used only to organize the included literature. The integrated framework identifies PD-1/PD-L1 as the dominant mechanistic hub linking T-cell activation, endothelial recruitment, myocardial injury, and vascular inflammation. Across phenotypes, a shared immune core involving checkpoint pathways, cytokine signaling, and leukocyte trafficking coexists with phenotype-restricted mediators that may bias injury toward myocarditis, vascular inflammation, conduction-system disease, or NILVD. KEGG analyses support the enrichment of T-cell receptor signaling, Th17 differentiation, JAK-STAT signaling, cytokine–cytokine receptor interaction, and lipid and atherosclerosis pathways. Candidate biomarkers emerging from the reviewed literature include troponin, IL-6, CXCL9/CXCL10/CXCL13, S100A family proteins, ROCK2, HLA-linked susceptibility signals, and T-cell receptor clonality markers. The AI-assisted clustering broadly recapitulated the expert-defined thematic structure while identifying finer semantic neighborhoods within the literature. This framework provides a support map for further hypotheses about toxicity patterns with current and next-generation checkpoint strategies on the cardiac system, while AI-assisted clustering provides a complementary method for organizing the literature rather than an independent source of biological inference.

## 1. Introduction

Immune checkpoint inhibitors (ICIs) have reshaped systemic cancer therapy over the past decade. Regulators in North America, Europe, and Asia have approved more than 15 agents targeting cytotoxic T-lymphocyte-associated protein-4 (CTLA-4), programmed cell death-1 (PD-1), programmed death-ligand 1 (PD-L1), and lymphocyte-activation gene 3 (LAG-3), now used as single drugs or in combination across many solid and hematologic cancers [1]. Beyond these established targets, a second wave of inhibitory receptors—including T-cell immunoreceptor with Ig and ITIM domains (TIGIT), T-cell immunoglobulin and mucin domain-containing protein 3 (TIM-3), V-domain Ig suppressor of T cell activation (VISTA), and B- and T-lymphocyte attenuator (BTLA)—is being actively explored, mostly in combination with PD-1/PD-L1 inhibitors.

In this context, previous research has established that myocarditis is the most widely recognized life-threatening cardiovascular-immune-related adverse event (irAE) among ICIs in routine oncology practice. Some studies indicate that ICI-related myocarditis has a reported incidence of 0.04% to 1.14% [2], while others suggest that myocarditis has an incidence ranging from 0.06% to 2.4% [3]. However, recent meta-analyses of randomized trials and large pharmacovigilance datasets place the incidence of ICI-associated myocarditis in the range of 0.3% to 1%, with significant differences between trial and real-world settings [4].

Hence, while myocarditis remains the archetypal and best-characterized cardiovascular irAE of ICIs, it is now clear that this represents only one phenotype of a broader spectrum of toxicities. Cohorts describe a wide range of cardiac manifestations—including atrial and ventricular arrhythmias, non-inflammatory left ventricular dysfunction (NILVD), heart failure, pericardial disease, vasculitis, and thrombo-ischemic events—many of which occur in the absence of a diagnostic biopsy or cardiovascular magnetic resonance, with observed myocardial inflammation [5,6]. These phenotypes have become much more evident as cardio-oncology hospitals adopt systematic surveillance and as follow-up extends beyond the early cycles of ICI therapy.

Mechanistically, hypotheses suggest that ICI-related cardiac dysfunction can manifest through microvascular dysfunction, stress-cardiomyopathy-like patterns, cytokine-mediated myocardial depression, or the unmasking of subclinical cardiomyopathy rather than frank autoimmune myocarditis [6,7]. Additionally, vasculitis, a non-myocarditis phenotype, has gained attention due to an association with ICIs. Systematic reviews and scoping analyses indicate that ICI-associated vasculitis is rare but highly heterogeneous, including large-vessel aortitis and giant-cell arteritis-like syndromes, medium-vessel vasculitis, cutaneous small-vessel vasculitis, and central nervous system vasculitis [8]. Furthermore, clinical and experimental data indicate that ICIs can accelerate the process of atherosclerosis. ICI exposure was associated with a significantly increased risk of atherosclerotic cardiovascular events, including myocardial infarction, stroke, and peripheral arterial events, compared to matched controls; these events clustered after ICI initiation [9,10]. Imaging studies and registry analyses confirmed that ICI therapy is associated with the accelerated progression of non-calcified plaque volume and increased rates of atherosclerotic events. This may occur due to immune activation within established plaques [11,12].

Preclinical models show that checkpoint inhibition exacerbates plaque inflammation and increases the atherosclerotic burden in mice, supporting a causal relationship [13]. However, there has been insufficient attention to the mechanistic process of these cardio-specific toxicities. Attempts to address mechanisms have been partial and fragmented. The loss of peripheral tolerance, CD8^+^ T-cell-mediated cytotoxicity, and macrophage-rich inflammation have been defined and studied but softly integrated as a complete proposed pathophysiological process [14,15,16]. Moreover, authoritative commentaries have explicitly highlighted the gap between our growing list of clinical entities and an incomplete understanding of the underlying biology [17,18]. Immunology and basic cardiovascular science journals report PD-1/PD-L1-deficient and checkpoint-blockade mouse models, revealing that disruption of this axis amplifies T-cell responses to cardiac antigens, worsens myocarditis, and impairs recovery after myocardial injury [19,20,21]. Other experimental research identifies PD-1/PD-L1 as a regulator of post-infarct remodeling and atherosclerotic plaque inflammation, suggesting that the same pathway influences ischemic injury and chronic vascular disease [9,22]. Oncology-focused studies explore how checkpoint blockade influences T-cell clones and myeloid populations within tumors; occasionally, this is tested for off-tumor tissues such as the heart [16,23]. Crucial insights for understanding cardiovascular risk, such as the cell-type-specific expression of PD-L1 in cardiomyocytes and endothelium, or checkpoint-dependent modulation of plaque-resident T cells, remain largely disconnected from clinical cardio-oncology narratives. Moreover, mechanistic data are scattered across immunology, cardiology, and oncology, generated in various models using diverse endpoints, and rarely synthesized across genes involved, their molecular translation, cellular integration, and their relationship to tissue, organ, and clinical phenotypes. This lack of an integrative mechanistic model obstructs our ability to identify patients at the highest risk a priori, rationally design surveillance strategies for myocarditis and non-myocarditis phenotypes, and anticipate the cardiovascular safety profiles of next-generation combinations targeting emerging checkpoints beyond PD-1/PD-L1 and CTLA-4.

The primary objective of this review is to explore the framework linking ICI exposure in cancer to cardiovascular phenotypes, including myocarditis, pericardial disease, conduction abnormalities, NILVD, vasculitis, and accelerated atherosclerosis. The secondary objectives are: (i) to map the recurrent genes, molecules, cell populations, tissues, and clinical phenotypes reported across the mechanistic literature; (ii) to compare shared and phenotype-restricted mediators through overlap analyses; (iii) to contextualize curated gene sets using KEGG pathway enrichment; (iv) to construct an integrated phenotype–molecule/gene network; and (v) to apply AI-assisted semantic clustering as an exploratory literature organization tool and compare it with an expert-curated taxonomy. Biological interpretation and pathway synthesis were performed by the authors; the AI component was used only for article organization, not for independent mechanistic inference.

## 2. Global Network Mechanisms

### 2.1. Gene Network-Level

**Figure 1:**
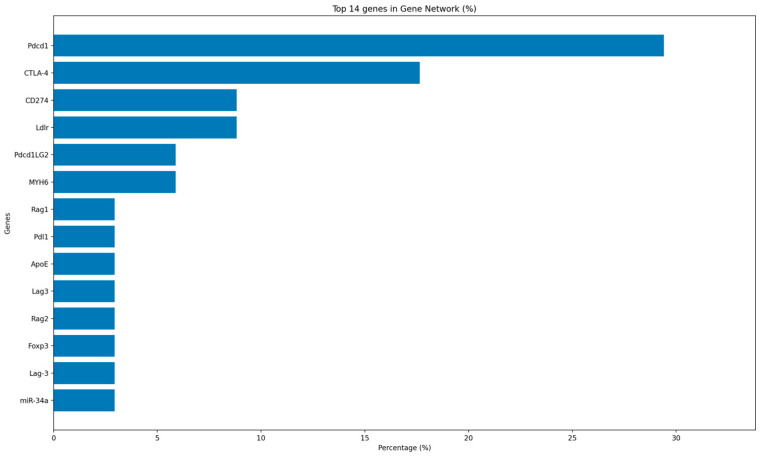
Most genes involved in ICI cardiac toxicities. Horizontal bars indicate the percentage of times each gene appears across the compiled interactions and pathways. Programmed cell death 1 (Pdcd1, PD-1) and cytotoxic T-lymphocyte-associated protein 4 (CTLA4, CTLA-4) are the most frequently represented nodes, followed by programmed cell death 1 ligand 1 (CD274, PD-L1), low-density lipoprotein receptor (Ldlr), programmed cell death 1 ligand 2 (Pdcd1LG2, PD-L2), myosin heavy chain 6 (MYH6), lymphocyte activation gene 3 (LAG3, Lag-3), apolipoprotein E (APOE), recombination activating gene 1 (RAG1), recombination activating gene 2 (RAG2), forkhead box P3 (FOXP3), and microRNA-34a (miR-34a).

Across all included studies, as expected, we identified a highly checkpoint-centric gene network. The highest proportional representation gene was PDCD1 (PD-1), followed by CTLA4. Together, these two co-inhibitory receptors formed the dominant axis of the gene network, reflecting that most mechanistic work to date has focused on PD-1/PD-L1 and CTLA-4 blockade. Checkpoint ligands were also notably represented: CD274 (PD-L1) and PDCD1LG2 (PD-L2) each appeared in multiple studies, often in conjunction with PDCD1, as is consistent with a recurrent PD-1/PD-L1/PD-L2 axis across phenotypes. Beyond immune checkpoints, we observed recurrent involvement of lipid-handling and structural genes, primarily LDLR and APOE, and the cardiomyocyte contractile gene MYH6 (α-myosin heavy chain). These genes bridge systemic immune modulation with local effects on lipoprotein metabolism and cardiomyocyte injury. Less frequently reported but biologically important nodes included RAG1/RAG2 involved in lymphocyte development, FOXP3 for regulatory T-cell lineage, and LAG3, indicating that perturbation of T-cell differentiation and additional inhibitory receptors may shape susceptibility to cardiac irAEs. Finally, miR-34a, a post-transcriptional regulator of gene expression, appeared as a non-coding regulator linked to cardiomyocyte stress and remodeling (Figure 1).

The gene-level map highlights that current mechanistic evidence is heavily centered on PD-1/CTLA-4 signaling, with limited data on other genes and transcription factors involved in the development of these cardiac irAEs. However, emerging signals in lipid metabolism, myocardial structural proteins, and T-cell differentiation pathways are under investigation.

### 2.2. Molecular Network-Level

**Figure 2:**
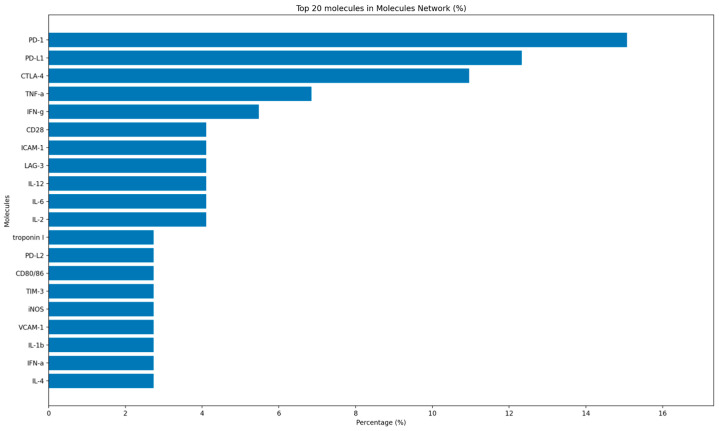
Top 20 molecules involved in the checkpoint–cardiovascular molecular network. Horizontal bars show the percentage at which each molecule appears across the data. The dominant nodes are the immune checkpoint receptors and ligands programmed cell death protein 1 (PD-1) and programmed death-ligand 1 (PD-L1), followed by cytotoxic T-lymphocyte-associated protein 4 (CTLA-4). Key inflammatory cytokines include tumor necrosis factor-alpha (TNF-α), interferon-gamma (IFN-γ), interleukin-12 (IL-12), interleukin-6 (IL-6), interleukin-2 (IL-2), interleukin-1 beta (IL-1β), interferon-alpha (IFN-α) and interleukin-4 (IL-4). Co-stimulatory and co-inhibitory receptors are CD28, lymphocyte activation gene-3 (LAG-3), T-cell immunoglobulin and mucin-domain-containing protein-3 (TIM-3), and the B7 family ligands CD80/86, as well as programmed death-ligand 2 (PD-L2). Vascular adhesion and injury-related molecules include intercellular adhesion molecule-1 (ICAM-1), vascular cell adhesion molecule-1 (VCAM-1), inducible nitric oxide synthase (iNOS) and the cardiomyocyte injury marker troponin I.

The molecular network further emphasizes the scientific focus on checkpoint pathways in cardiac irICI. The highest proportional reported molecular entities were PD-1, PD-L1, and CTLA-4. These three molecules form the core axis of current clinical immune checkpoint blockade. Immediately downstream of checkpoints, the network was dominated by pro-inflammatory cytokines and effector molecules, including TNF-α, IFN-γ, IL-12, IL-6, and IL-2. IFN-γ is produced mainly by activated Th1-polarized CD4^+^ T cells, cytotoxic CD8^+^ T cells, and NK cells, where it promotes macrophage activation and enhances antigen presentation within inflamed myocardium. TNF-α is derived largely from monocyte-derived macrophages but can also be released by activated T cells and NK cells; it drives endothelial activation, leukocyte recruitment, and cardiomyocyte stress signaling. IL-12 is secreted primarily by antigen-presenting cells, such as dendritic cells and macrophages, and it reinforces Th1 polarization and IFN-γ production, thereby sustaining cytotoxic effector programs. IL-6 is generated chiefly by myeloid cells and can also be produced by activated endothelium and cardiac stromal cells under inflammatory stress, supporting acute-phase responses and further leukocyte trafficking. Finally, IL-2 is produced predominantly by activated T cells and serves as a key autocrine and paracrine growth factor that expands effector T-cell populations, increasing the likelihood of tissue injury once cardiac tolerance checkpoints are removed. Checkpoint-related co-stimulatory interactions such as CD28 and CD80/86 also appeared among the top-ranked molecules. Endothelial and leukocyte adhesion markers observed were ICAM-1 and VCAM-1, with vascular and myocardial levels consistent with enhanced leukocyte recruitment and microvascular inflammation. Additional inhibitory receptors LAG-3 and TIM-3, and effector enzymes such as iNOS, appeared but were less frequent. Troponin I was captured as a molecular injury marker, recurrently used to define myocarditis and NILVD in clinical reports, linking the mechanistic network to clinically measurable endpoints (Figure 2).

Overall, the molecular map reveals that the PD-1 and CTLA-4 axis and their ligands still represent a major focus in current literature. However, due to their limitations in fully explaining the pathophysiological process of cardiac irAE, several new targets have been developed: (1) pro-inflammatory cytokines and effector molecules; (2) checkpoint-related co-stimulatory molecules; (3) adhesion markers; (4) enzymes and cardiac proteins.

### 2.3. Cellular Network-Level

**Figure 3:**
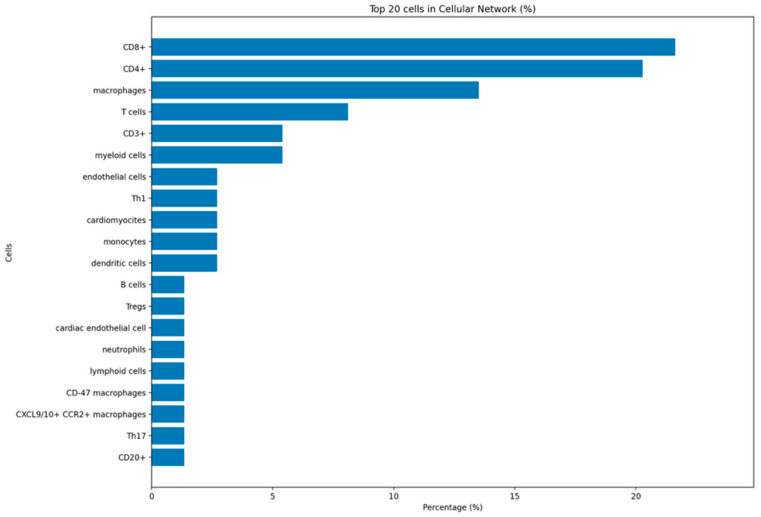
Top 20 recurrent cell populations in the curated checkpoint–cardiovascular cellular network. Horizontal bars show the percentage each cell type appears across the compiled experimental and clinical studies. The network is dominated by CD8^+^ cytotoxic T lymphocytes (CD8^+^ T cells) and CD4^+^ helper T lymphocytes (CD4^+^ T cells), followed by macrophages, non-specified T cells and CD3^+^ T cells (CD3^+^ pan–T cells). Additional frequently represented populations include myeloid cells, endothelial cells, T helper type 1 cells (Th1), cardiomyocytes, monocytes, dendritic cells, and B cells. Regulatory T cells (Tregs), cardiac endothelial cells, neutrophils, and broader lymphoid cells also recur. More specialized subsets comprise CD47-expressing macrophages (CD47^+^ macrophages), CXCL9/10-producing, CCR2-expressing macrophages (CXCL9/10^+^ CCR2^+^ macrophages), T helper 17 cells (Th17), and CD20^+^ B cells (CD20^+^ B lymphocytes).

The cellular layer of the network was dominated by effector T cells and macrophages. The highest proportional reported populations were CD8^+^ T cells and CD4^+^ T cells, each described in the majority of mechanistic studies, followed closely by macrophages and generic T cells. CD3^+^ T cells and myeloid cells also appeared repeatedly, emphasizing that cardiac irAE studies shift their focus to a T-cell and myeloid-driven process rather than a purely humoral phenomenon. Among tissue-resident or target cells, endothelial cells and cardiomyocytes were the most commonly studied, often in conjunction with checkpoint ligand expression such as PD-L1 on the endothelium or as targeted by cytotoxic CD8^+^ T cells. Monocytes, dendritic cells, and B cells formed a secondary node of innate and adaptive immune populations, reflecting their roles in antigen presentation, costimulation, and antibody production. Regulatory compartments were also represented: multiple studies reported Tregs (Foxp3^+^) and regulatory B cells, albeit less frequently than effector subsets. More granular subpopulations—including Th1 and Th17 CD4^+^ T cells, CXCL9/10^+^ CCR2^+^ or CD47^+^ macrophages, CD68^+^ macrophages, and CAR T cells in the context of engineered T-cell therapies—were described in a smaller number of experimental or clinical studies (Figure 3).

Together, the cellular network highlights that most of the studies in the current literature show that the checkpoint blockade stimulates CD8^+^ and CD4^+^ effector T cells and pro-inflammatory macrophages at the interface of endothelium and cardiomyocytes. However, a shift in mechanistic approaches is underway, with variable contributions from dendritic cells, neutrophils, B cells, and regulatory subsets shaping the eventual phenotype (myocarditis, vasculitis, conduction disease, or atherosclerosis).

### 2.4. Organ-Level Network

**Figure 4:**
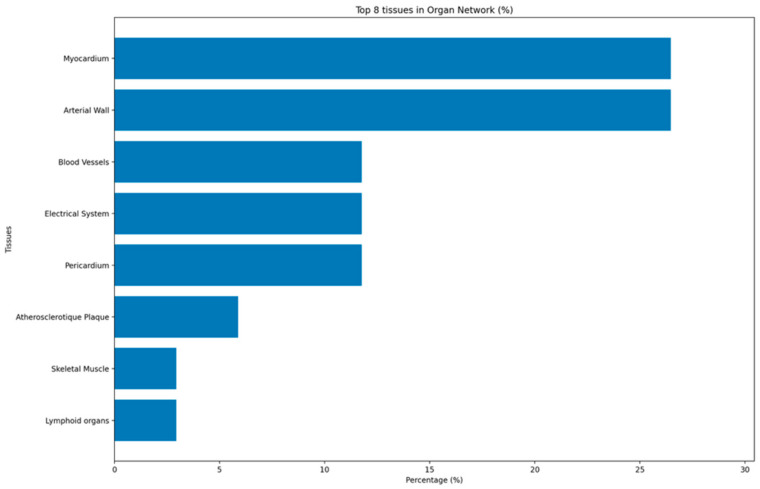
Top 8 recurrent tissues in the curated checkpoint–cardiovascular organ network. Horizontal bars indicate the percentage at which each tissue or anatomical compartment appears across the compiled studies. The myocardium and arterial wall are the most frequently represented sites, followed by blood vessels and the cardiac electrical system. The pericardium and atherosclerotic plaque are also prominent targets, while skeletal muscle and secondary lymphoid organs appear less often.

The organ-level network shows that mechanistic studies of ICI cardiotoxicity are condensed in a few key cardiovascular compartments. The myocardium and the arterial wall were the highest proportional examined tissues, each appearing in nine mechanistic reports. This reflects the dominance of myocarditis and accelerated atherosclerosis as index phenotypes in the current literature, underscoring that most experimental research focuses either on cardiomyocyte injury or on plaque-level vascular inflammation. A second tier of anatomical sites included blood vessels, the cardiac electrical system, and the pericardium, each reported in four studies. These tissues correspond to vasculitis, microvascular inflammation, conduction abnormalities, and pericardial disease, illustrating that checkpoint-related injury is not restricted to contractile myocardium but involves the vascular and electrophysiological architecture of the heart. Atherosclerotic plaque was explicitly specified as a distinct tissue in a smaller number of studies, typically in murine models of plaque progression under checkpoint modulation. Less frequently sampled tissues include skeletal muscle and lymphoid organs, usually examined either as comparators for cardiac involvement (to distinguish myocarditis from myositis) or as sites of immune priming and checkpoint expression such as the lymph nodes and spleen (Figure 4).

Overall, the tissue network indicates that available mechanistic evidence is heavily weighted toward myocardium and arterial wall, with fewer data on conduction tissue, microvasculature, and extra-cardiac immune organs.

### 2.5. Phenotype Network

**Figure 5:**
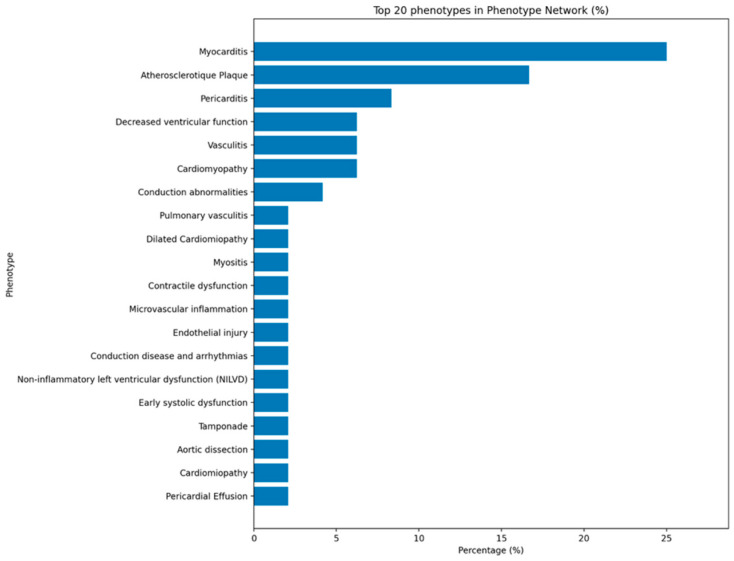
Top 17 recurrent cardiac toxicities in the curated checkpoint–cardiovascular phenotype network. Horizontal bars indicate the percentage at which each clinical phenotype is reported across the compiled immune checkpoint inhibitor studies. Myocarditis is the most frequently represented event, followed by progression or destabilization of atherosclerotic plaque and cardiomyopathy. Other common phenotypes include decreased ventricular function, vasculitis involving cardiac or great-vessel territories, and pericarditis. Less studied phenotypes include conduction abnormalities and pulmonary vasculitis. Additional toxicities presented in the network are dilated cardiomyopathy, myositis, microvascular inflammation, endothelial injury, and non-ischemic left ventricular dysfunction (NILVD), as well as early systolic dysfunction, cardiac tamponade, aortic dissection, and pericardial effusion.

The phenotypic network shows that mechanistic research on ICI cardiotoxicity is heavily concentrated on a limited set of cardiovascular clinical spectrum. Myocarditis was by far the most frequently reported phenotype, appearing in roughly one-third of all included mechanistic records and clearly dominating the spectrum. Atherosclerotic plaque and accelerated atherosclerosis were the second most common entities, followed by pericarditis, reduced ventricular function, vasculitis, and conduction abnormalities. These six categories together account for the great majority of mechanistic descriptions. A larger group of less frequently reported outcomes included microvascular inflammation, endothelial injury, pulmonary vasculitis, contractile dysfunction, tamponade, pericardial effusion, thrombosis, vascular leak, ventricular tachycardia, heart block, cardiogenic shock, and sudden death, most of which were encountered only once or twice in the mechanistic literature we used for screening (Figure 5).

The distribution indicates that current mechanistic understanding of cardiac irICI is grounded primarily in myocarditis and atherosclerotic disease, whereas conduction disease, microvascular injury, and thrombotic/vascular leak phenotypes are recognized but are supported by comparatively sparse mechanistic data.

Taken together, these descriptive network layers indicate that the current mechanistic literature is heavily centered on a checkpoint-driven immune core involving PD-1/PD-L1, CTLA-4, effector T cells, macrophages, endothelial activation, and the myocardium–arterial wall axis. At the same time, the imbalance in the literature is evident: myocarditis and atherosclerosis dominate the available mechanistic evidence, whereas conduction disease, NILVD, microvascular injury, and several vascular phenotypes remain underrepresented. For this reason, the following sections focus less on entity frequency alone and more on how shared versus phenotype-specific mediators can be integrated into a unified mechanistic framework.

## 3. Phenotypic Overlap Analysis

### 3.1. Gene-Level Overlap Across Phenotypes

To explore whether ICI-related cardiovascular toxicities share a common mechanistic scaffold or instead remain largely phenotype-specific, we compared curated gene, molecular, and cell-level datasets across the major phenotypes. These overlap analyses were intended to identify both a shared immune core and phenotype-restricted mediators. Because numerical overlap alone can be difficult to interpret, the identities of key shared and unique mediators are additionally summarized in the Supplementary File S1. The goal of this section is therefore not only to quantify overlap, but also to clarify which biological programs are common to multiple phenotypes and which may bias injury toward myocarditis, vascular inflammation, conduction-system disease, or NILVD.

**Figure 6:**
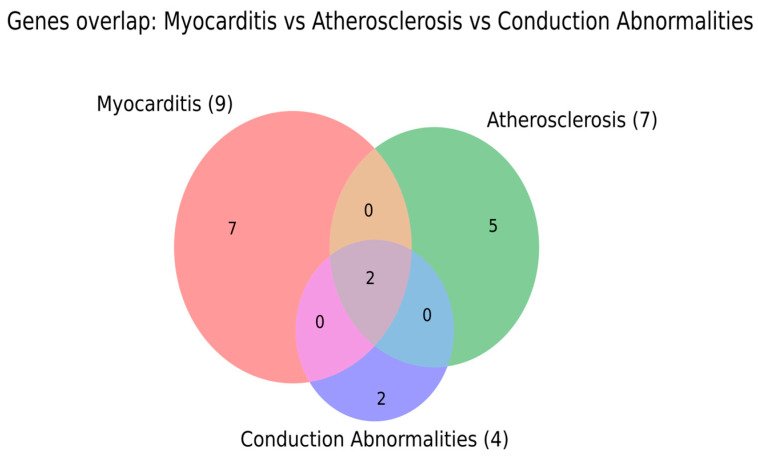
Gene overlap between myocarditis, atherosclerosis and conduction abnormalities in the molecular network. Three-way Venn diagram depicting the number of curated genes associated with immune checkpoint inhibitor–related myocarditis (red, 9 genes), atherosclerosis-related events (green, 7 genes) and conduction abnormalities (blue, 4 genes). Numbers within each sector indicate the count of genes unique to a given phenotype or shared across phenotypes. Myocarditis, atherosclerosis and conduction abnormalities show predominantly distinct gene sets (7, 5 and 2 unique genes, respectively), with only 2 genes shared by all three phenotypes and no pairwise-only overlaps.

We compared gene sets curated for myocarditis (nine genes), atherosclerosis-related events (seven genes), and conduction abnormalities (four genes). The Venn diagram shows that each phenotype retains a largely distinct gene signature, with seven genes unique to myocarditis, five unique to atherosclerosis, and two unique to conduction abnormalities. Only two genes are shared across all three phenotypes, and there are no genes shared exclusively between any pair of phenotypes (myocarditis–atherosclerosis, myocarditis–conduction, or atherosclerosis–conduction) (Figure 6).

Biologically, the limited gene-level overlap suggests that the available literature may not yet define a broad, complete, shared genetic program across myocarditis, atherosclerosis, and conduction abnormalities. The small common core may represent upstream checkpoint-related immune regulation, whereas the phenotype-restricted genes likely reflect tissue-specific injury contexts: cardiomyocyte antigenicity and myocardial immune infiltration in myocarditis, lipid handling and plaque biology in atherosclerosis, and incompletely characterized immune-electrophysiological mechanisms in conduction disease. However, the absence of pairwise-only overlap should not be interpreted as proof that these phenotypes are genetically independent. It may also reflect uneven study depth, with myocarditis and atherosclerosis studied more extensively than conduction-system toxicity. Clinically, this supports caution in extrapolating myocarditis-derived genetic or molecular biomarkers to conduction abnormalities or vascular events without phenotype-specific validation.

### 3.2. Molecule-Level Overlap Across Phenotypes

**Figure 7:**
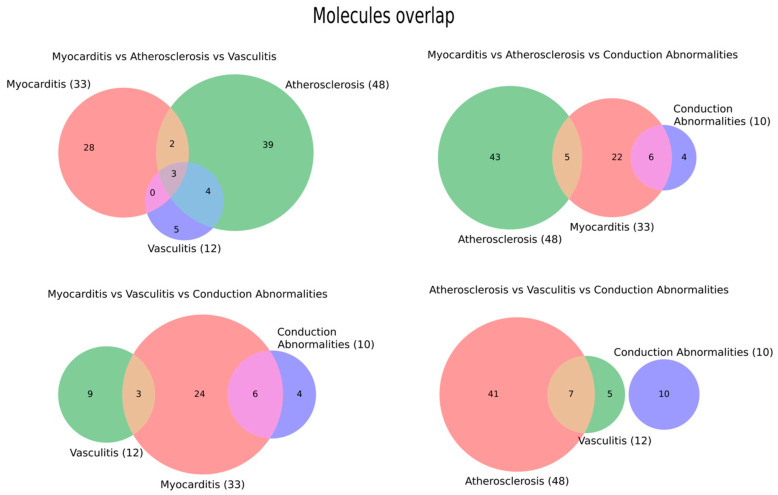
Overlap of inflammatory mediators across myocarditis, atherosclerosis, vasculitis and conduction abnormalities. Four triple Venn diagrams summarize the distribution of curated molecules for (**upper left**) myocarditis (33 molecules), atherosclerosis (48 molecules) and vasculitis (12 molecules); (**upper right**) myocarditis, atherosclerosis and conduction abnormalities (10 molecules); (**lower left**) myocarditis, vasculitis and conduction abnormalities; and (**lower right**) atherosclerosis, vasculitis and conduction abnormalities. Numbers in each segment indicate the count of molecules unique to a given phenotype or shared between two or three phenotypes.

We next examined how inflammatory mediators are shared across the major immune-checkpoint-inhibitor-associated cardiovascular phenotypes by constructing triple Venn diagrams for myocarditis, atherosclerosis, vasculitis, and conduction abnormalities. In the myocarditis–atherosclerosis–vasculitis triad (upper left), each phenotype retained a large set of unique molecules (28 for myocarditis, 39 for atherosclerosis, and 5 for vasculitis), with only 2 molecules shared by myocarditis and atherosclerosis, 4 shared by atherosclerosis and vasculitis, none shared exclusively by myocarditis and vasculitis, and a small three-way core of 3 molecules common to all three phenotypes. In contrast, the myocarditis–atherosclerosis–conduction abnormalities triad (upper right) showed extensive uniqueness (22, 43 and 4 molecules, respectively) and only pairwise overlap between myocarditis and atherosclerosis (5 molecules) or myocarditis and conduction abnormalities (6 molecules), with no molecules shared between atherosclerosis and conduction abnormalities and no three-way intersection. The myocarditis–vasculitis–conduction abnormalities triad (lower left) was similarly dominated by phenotype-specific mediators (24 for myocarditis, 9 for vasculitis, and 4 for conduction abnormalities), with limited pairwise sharing between myocarditis and vasculitis (3 molecules) and between myocarditis and conduction abnormalities (6 molecules), and no molecules common to all three. Finally, in the atherosclerosis–vasculitis–conduction abnormalities triad (lower right), atherosclerosis and vasculitis shared 7 molecules, while vasculitis, and conduction abnormalities and the three-way overlap each contained no molecules, leaving 41, 5 and 10 unique mediators for atherosclerosis, vasculitis and conduction abnormalities, respectively (Figure 7).

At the molecular level, the overlap pattern is more informative for pathological stratification. Molecules shared among myocarditis, atherosclerosis, and vasculitis may represent a common inflammatory backbone involving checkpoint disruption, T-cell activation, cytokine amplification, and endothelial recruitment. This shared module may explain why clinically distinct phenotypes can emerge under the same therapeutic exposure to PD-1/PD-L1 or CTLA-4 blockade. In contrast, the large phenotype-restricted molecular fractions may suggest that tissue context modifies the final injury pattern: myocardial antigens and cardiomyocyte stress pathways may bias toward myocarditis, adhesion molecules and vascular inflammatory mediators toward vasculitis, and lipid/plaque-associated mediators toward accelerated atherosclerosis. The limited overlap involving conduction abnormalities should be interpreted cautiously. It may indicate a genuinely distinct electrophysiological injury module, but it may also reflect how conduction disease is usually reported clinically as part of myocarditis rather than studied as an independent mechanistic phenotype. From a diagnostic perspective, these findings argue against assuming that one circulating inflammatory panel will perform equally across all ICI cardiovascular toxicities.

**Figure 8:**
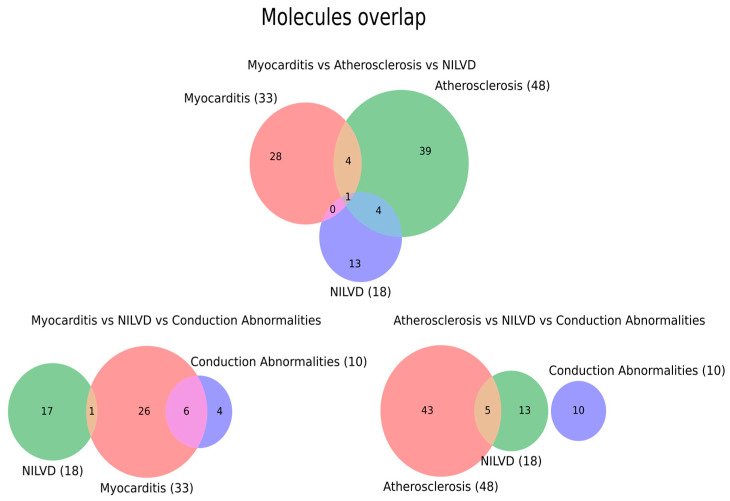
Molecular overlap between myocarditis, atherosclerosis, NILVD, and conduction abnormalities. Three triple Venn diagrams display the distribution of curated inflammatory mediators for (**upper**) myocarditis (33 molecules), atherosclerosis (48 molecules) and NILVD (18 molecules); (**lower**) myocarditis, atherosclerosis and conduction abnormalities (10 molecules); (**lower left**) myocarditis, NILVD and conduction abnormalities; and (**lower right**) atherosclerosis, NILVD and conduction abnormalities. Numbers in each region indicate the count of molecules unique to a given phenotype or shared between two or three phenotypes.

We next asked how NILVD fits into the molecular landscape of immune checkpoint-inhibitor-associated cardiovascular toxicity by comparing its mediator set with those of myocarditis, atherosclerosis and conduction abnormalities. In the myocarditis–atherosclerosis–NILVD triad, each phenotype preserved a large number of unique molecules (28, 39 and 13, respectively), with modest pairwise overlap between myocarditis and atherosclerosis (4 molecules) and between atherosclerosis and NILVD (4 molecules), no molecules shared exclusively by myocarditis and NILVD, and a single molecule common to all three phenotypes. The myocarditis–NILVD–conduction abnormalities triad showed similar structure, with 26, 17 and 4 unique molecules, only 1 molecule shared between myocarditis and NILVD and 6 shared between myocarditis and conduction abnormalities. Finally, in the atherosclerosis–NILVD–conduction abnormalities triad, atherosclerosis and NILVD shared 5 molecules, whereas NILVD and conduction abnormalities, as well as the three-way intersection, contained no common mediators (43, 13 and 10 unique molecules, respectively) (Figure 8).

The inclusion of NILVD further supports the need for pathological stratification. In our analysis, NILVD showed only limited molecular overlap with myocarditis and atherosclerosis and minimal overlap with conduction abnormalities, suggesting that it should not be automatically interpreted as subclinical or biopsy-negative myocarditis. Instead, NILVD may represent a partially distinct injury phenotype involving cytokine-mediated myocardial depression, microvascular dysfunction, stress-cardiomyopathy-like mechanisms, or unmasking of pre-existing cardiac vulnerability. This distinction has practical diagnostic implications: troponin-centered myocarditis algorithms may be insufficient for NILVD, where echocardiography, cardiac magnetic resonance, longitudinal ventricular function assessment, and heart failure biomarkers may be more informative. At the same time, the NILVD literature remains sparse, so non-overlap may partly reflect underreporting and inconsistent phenotype definitions rather than true mechanistic independence.

### 3.3. Cell-Level Overlap Across Phenotypes

**Figure 9:**
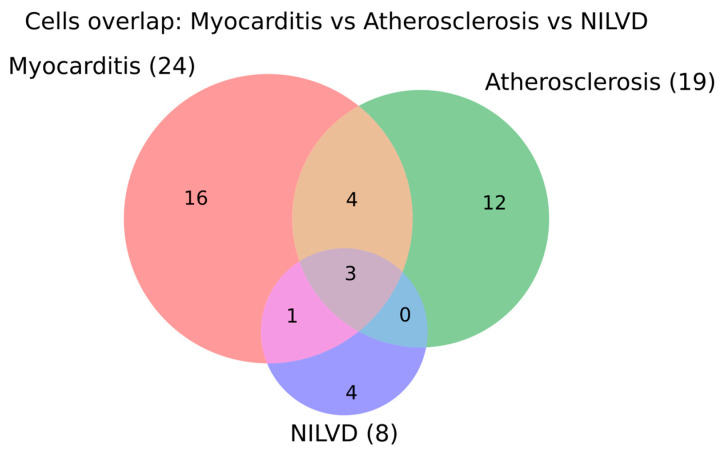
Overlap of cellular populations across myocarditis, atherosclerosis and non-ischemic left ventricular dysfunction (NILVD). Three-set Venn diagram depicting the number of curated cell populations associated with myocarditis (red, 24 cell types), atherosclerosis (green, 19 cell types) and NILVD (blue, 8 cell types). Numbers within each region correspond to cell populations unique to a single phenotype or shared between two or all three phenotypes. Myocarditis, atherosclerosis and NILVD each retain substantial unique cellular entities, with only a small three-way core of 3 shared cell populations.

To examine whether similar cellular effectors are discussed across different immune-checkpoint-inhibitor-associated phenotypes in the current literature, we compared the cell populations annotated for myocarditis, atherosclerosis and NILVD. The Venn diagram shows that each phenotype is dominated by a distinct cell bank, with 16 cell populations unique to myocarditis, 12 unique to atherosclerosis and 4 unique to NILVD. Overlap is modest: myocarditis and atherosclerosis share 4 cell populations, myocarditis and NILVD share only 1, and there are no cell types shared exclusively between atherosclerosis and NILVD. A small core of three cell populations is common to all three phenotypes (Figure 9).

At the cellular level, the small shared core across myocarditis, atherosclerosis, and NILVD likely reflects a common immune-effector backbone composed of T-cell and myeloid activation. However, the phenotype-restricted cellular compartments suggest different tissue-level execution mechanisms. In myocarditis, cytotoxic and helper T-cell infiltration with macrophage-rich inflammation remains the dominant model. In atherosclerosis, vascular wall macrophages, T cells, endothelial cells, and smooth-muscle-derived inflammatory cell states are more relevant to plaque progression and destabilization. In NILVD, the smaller and more distinct cellular set may indicate mechanisms less dependent on dense inflammatory infiltrates, such as cytokine-mediated contractile dysfunction, microvascular dysfunction, or stress-related myocardial impairment. These differences support phenotype-specific surveillance: myocarditis requires early injury-marker and rhythm monitoring, atherosclerosis may require vascular risk assessment and imaging in selected patients, and NILVD requires longitudinal ventricular-function monitoring. Nevertheless, because NILVD and conduction-system injury remain underrepresented in mechanistic studies, the apparent cellular separation should be considered hypothesis generating.

## 4. KEGG Pathway Enrichment Analysis

### 4.1. KEGG Pathway Enrichment of the Global Molecular Network

**Figure 10:**
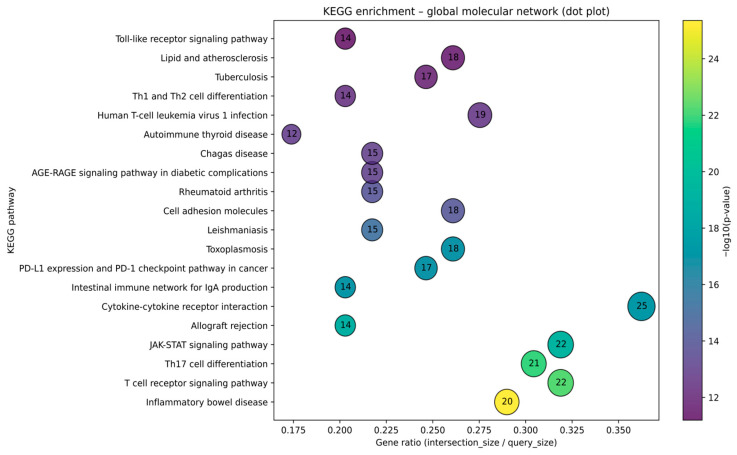
Dot-plot representation of KEGG enrichment for the global checkpoint–cardiovascular molecular network. Each dot represents a KEGG pathway enriched by hypergeometric over-representation analysis. The *x*-axis shows the gene ratio, the *y*-axis lists KEGG pathways, dot size is proportional to the number of overlapping genes (k; value shown inside each dot), and dot color encodes statistical significance as the negative logarithm of the *p*-value (−log_10_p).

Dot-plot visualization of the KEGG over-representation analysis for the global checkpoint–cardiovascular molecular network confirms a dense enrichment of adaptive and innate immune pathways. The gene ratio shows that “Inflammatory bowel disease,” “T cell receptor signaling pathway,” “Th17 cell differentiation,” and “JAK–STAT signaling pathway” not only have highly significant *p*-values but also capture the largest fractions of the network, with gene ratios around 0.30–0.36 and overlaps of 20–22 genes. “Cytokine–cytokine receptor interaction” shows the highest absolute overlap (k = 25) despite a slightly lower gene ratio, emphasizing its role as one of the most recurrent enriched signaling categories. Autoimmune and alloimmune pathways—“Allograft rejection,” “Rheumatoid arthritis,” “Autoimmune thyroid disease,” and the “PD-L1 expression and PD-1 checkpoint pathway in cancer”—cluster in the mid-to-high gene ratio and significance range, indicating that the curated network strongly reuses molecular programs characteristic of systemic autoimmunity and checkpoint biology found in the current literature. Infectious and innate immune pathways such as toxoplasmosis, leishmaniasis, tuberculosis, Chagas disease, human T-cell leukemia virus 1 infection, and the “Toll-like receptor signaling pathway” form an adjacent cluster with moderate-to-high gene ratios, reflecting shared inflammatory pathways. Finally, “Lipid and atherosclerosis” and the “AGE–RAGE signaling pathway in diabetic complications” (advanced glycation end products–receptor for AGE) exhibit substantial gene ratios and significance, linking this checkpoint-centric network to vascular inflammation and metabolic atherogenesis. Moreover, the analysis exposed the “PD-L1 expression and PD-1 checkpoint pathway in cancer” pathway, confirming that our curated network faithfully captures the on-target pharmacologic module (Figure 10).

### 4.2. KEGG Enrichment by Phenotype

To improve our mechanistic approach and to help clarify organ-specific hypotheses in the current literature, we performed KEGG enrichment not only on the entire molecular network but also on subnetworks stratified by clinical phenotype (myocarditis, atherosclerosis, vasculitis). This phenotype-level analysis allows us to identify which pathways are shared versus selectively amplified across distinct forms of immune checkpoint inhibitor–associated cardiovascular toxicity. By resolving enrichment signatures at the level of individual phenotypes, we can distinguish generic immune pathways such as T-cell receptor signaling, Th17 differentiation, and cytokine–cytokine receptor interaction from pathways that appear preferentially connected into myocardial inflammation, vascular wall disease, or systemic vasculitis. This, in turn, provides a mechanistic rationale for organ-specific vulnerability, supports the existence of partially overlapping but distinct checkpoint-injury studies, and generates targeted hypotheses for biomarkers and therapeutic interventions tailored to each toxicity pattern rather than treating “ICI cardiotoxicity” as a single homogeneous entity.

**Figure 11:**
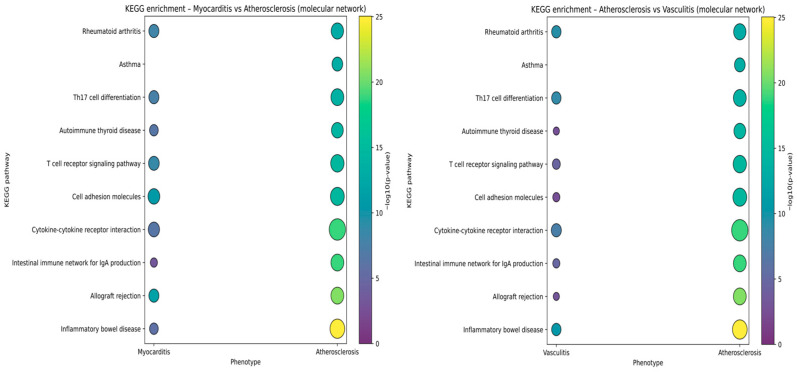
KEGG pathway enrichment for myocarditis, atherosclerosis and vasculitis molecular subnetworks. Dot plots show hypergeometric KEGG over-representation for (**left**) myocarditis vs. atherosclerosis and (**right**) atherosclerosis vs. vasculitis. The *y*-axis lists selected KEGG pathways; the *x*-axis indicates the phenotype-specific molecular subnetwork (myocarditis, atherosclerosis or vasculitis). Each dot represents a pathway–phenotype pair; dot size is proportional to the number of overlapping genes, and color encodes statistical significance as the negative logarithm of the *p*-value (−log_10_p).

Comparative KEGG enrichment of phenotype-specific molecular subnetworks showed that myocarditis, atherosclerosis, and vasculitis contain a common set of adaptive immune and autoimmune pathways, but with different strengths of association. For both comparisons, atherosclerosis displayed the most pronounced enrichment, with higher −log_10_(p) values across “inflammatory bowel disease,” “T cell receptor signaling pathway,” “T helper 17 (Th17) cell differentiation,” “cytokine–cytokine receptor interaction,” and “cell adhesion molecules,” indicating that the atherosclerotic module is densely wired into systemic autoimmune and T-cell signaling programs. Myocarditis and vasculitis showed concordant but weaker enrichment for these same pathways, with more modest signals for the “intestinal immune network for IgA production,” “allograft rejection,” “autoimmune thyroid disease,” and “asthma.” These patterns may suggest that all three phenotypes share a core autoimmune/Th17–T cell receptor signaling backbone, while the atherosclerotic network exhibits a larger integration of these immune pathways (Figure 11).

Importantly, these enriched KEGG terms should not be interpreted as uniquely cardiac signatures. Rather, they define a shared inflammatory scaffold that is common to multiple immune-mediated conditions and becomes relevant to irCV adverse reactions only when deployed within specific cardiovascular compartments. In our framework, mechanistic specificity arises less from pathway exclusivity than from where and how these pathways are expressed: myocardial injury may more plausibly be linked to cardiomyocyte-associated antigens, local PD-L1 loss, cytotoxic T-cell engagement, and stress/necrosis programs; vascular phenotypes are plausibly linked to endothelial activation, leukocyte adhesion, and VSMC phenotypic remodeling; and conduction-system disease likely reflects extension of immune injury into specialized myocardial tissue rather than a fully distinct immune pathway. Thus, the enriched pathways should be interpreted as context-dependent immune programs rather than phenotype-specific drivers in isolation.

## 5. Integrated Phenotype–Molecule/Gene Network

To integrate mechanistic information across phenotypes, we constructed a bipartite phenotype–molecule/gene network. Phenotype nodes (myocarditis, pericarditis, vasculitis, conduction abnormalities, atherosclerosis, NILVD) were placed centrally, and outer nodes corresponded to genes inferred from curated molecular entities.

**Figure 12:**
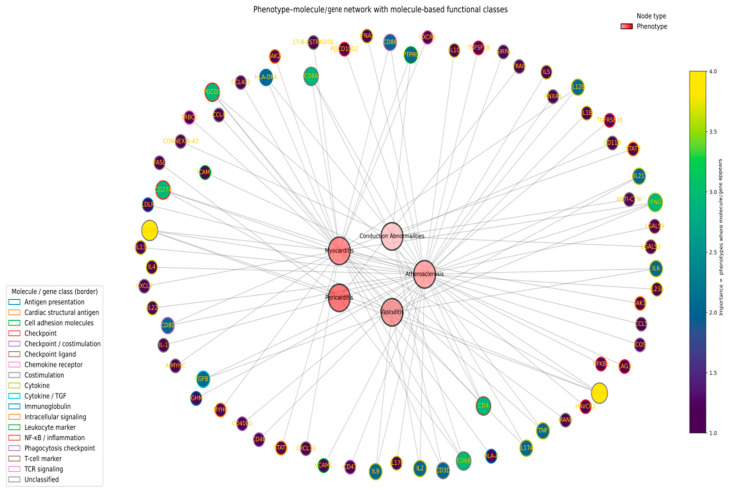
Phenotype–molecule/gene network annotated with molecule-based functional classes. Network layout showing cardiovascular phenotypes (red central nodes) connected to molecules (outer ring). Edges indicate literature-derived associations between a molecule and a given phenotype. The color of each molecular node encodes the number of phenotypes in which it appears (see color bar), whereas the node border color denotes its functional class.

For each gene node, we defined an “importance” score as the number of distinct phenotypes in which it appeared. This importance score was encoded in node fill color (viridis color scale) and partly in node size (larger nodes for genes shared by more phenotypes). In parallel, each molecular token was assigned to a functional class (checkpoint, checkpoint ligand, cytokine, chemokine receptor, cell adhesion molecule, complement, immunoglobulin, T-cell marker, macrophage marker, metabolic/mitochondrial protein, intracellular signaling, cardiac structural antigen), and these classes were aggregated to a dominant functional class per gene. Gene node borders were colored according to this functional class, allowing for the simultaneous visualization of “what the gene does” (border color) and “how many phenotypes it links” (fill intensity). Network layout was computed using a force-directed (spring) algorithm with phenotype nodes constrained in a small central ring, such that shared mediators are pulled into intermediate positions between phenotypes, whereas phenotype-restricted mediators remain closer to a single phenotype (Figure 12).

The phenotype–molecule network revealed mediators linking multiple immune-checkpoint-inhibitor-associated cardiovascular phenotypes, superimposed on a larger halo of phenotype-restricted molecules. Central phenotypes (myocarditis, atherosclerosis, vasculitis, pericarditis, and conduction abnormalities) were most frequently connected to checkpoint and costimulatory axes (CTLA-4, PD-1/PD-L1, CD28, CD40/CD40L, CD80/CD86), pro-inflammatory cytokines (TNF, IL-1 family, IL-6, IL-17A/F, IL-21, IL-23), and cell adhesion molecules (ICAM-1, VCAM-1), which formed a shared “immune core” present in three or more phenotypes. Molecules with high importance scores (appearing in ≥3 phenotypes) were enriched for functional classes related to T-cell receptor signaling, NF-κB/inflammatory signaling and leukocyte trafficking, supporting a model in which recurrent T-cell activation, cytokine amplification and endothelial activation reinforce diverse clinical presentations. In contrast, low-importance nodes (linked to a single phenotype) included cardiac structural antigens (myosin heavy chain isoforms), phagocytosis checkpoints, and selected chemokines/chemokine receptors, which clustered around specific phenotypes such as myocarditis or atherosclerosis. This pattern may show that checkpoint toxicities share a conserved immunological scaffold but are differentiated by additional, tissue-selective mediators that bias the response toward myocardial, vascular or conduction system injury (Figure 12).

**Figure 13:**
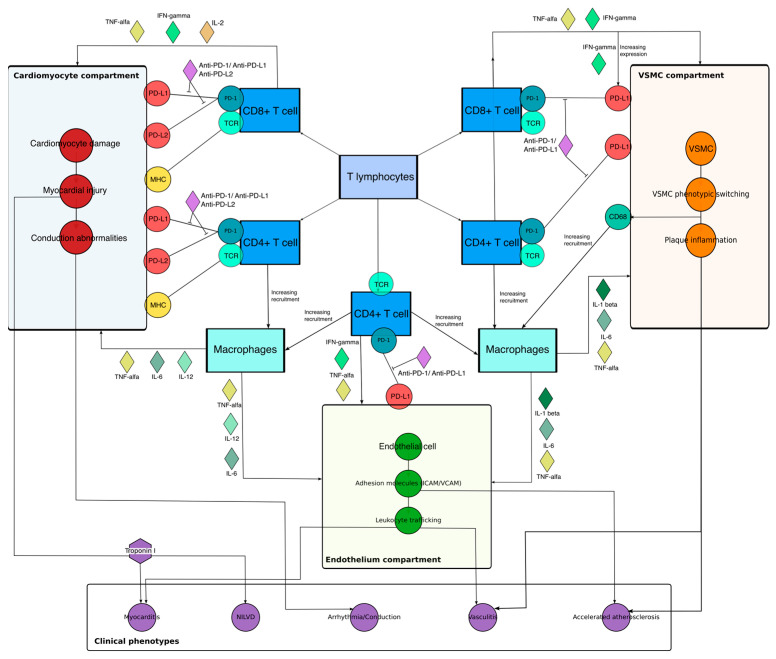
PD-1/PD-L1 network linking immune synapses to cardiac and vascular phenotypes. The diagram depicts three target compartments—cardiomyocyte (left), endothelium (bottom), and VSMC (right)—interacting with CD8^+^ and CD4^+^ T lymphocytes via TCR–MHC antigen recognition and PD-1 inhibitory signaling driven by PD-L1/PD-L2 at the level of membranes. Endothelial activation induces ICAM-1 and VCAM-1 upregulation and increases leukocyte trafficking, promoting macrophage and T-cell recruitment and microvascular inflammation. In the arterial wall, immune-driven signaling promotes VSMC phenotypic switching including CD68 expression and plaque inflammation, providing a mechanistic bridge to accelerated atherosclerosis. Clinical outputs summarized along the bottom include myocarditis, NILVD, conduction abnormalities, vasculitis, and accelerated atherosclerosis, with cTnI shown as a representative injury biomarker. cTnI: cardiac troponin I; ICAM-1: intercellular adhesion molecule-1; MHC: major histocompatibility complex; NILVD: non-inflammatory left ventricular dysfunction; PD-1: programmed cell death protein 1; PD-L1/PD-L2: programmed death-ligand 1/2; TCR: T-cell receptor; VCAM-1: vascular cell adhesion molecule-1; VSMC: vascular smooth muscle cell.

In mechanistic terms, the integrated model can be understood across three interacting compartments. In the cardiomyocyte compartment, antigen recognition and loss of inhibitory signaling at the PD-1/PD-L1 axis permit cytotoxic and helper T-cell engagement, promoting myocardial stress, necrosis, and clinically overt myocarditis or conduction-system injury. In the endothelial compartment, inflammatory cytokines and checkpoint dysregulation amplify ICAM-1/VCAM-1 expression, leukocyte adhesion, and tissue recruitment, thereby linking myocardial injury to vascular and microvascular inflammation. In the arterial wall and VSMC compartment, chronic inflammatory signaling and altered checkpoint tone promote VSMC activation, phenotypic switching, and plaque remodeling, providing a mechanistic bridge to accelerated atherosclerosis and selected vasculitic phenotypes. Thus, the proposed framework suggests that ICI-related cardiovascular toxicities share a conserved immune checkpoint–inflammation axis but diverge according to tissue context and phenotype-selective mediators (Figure 13).

## 6. Analysis of AI-Based Clustering

Artificial intelligence (AI) techniques are often used to group (cluster) items, such that items within each group are more similar to each other than to items from other groups. To discover such groups of research articles on ICI cardiac toxicities, we conducted an AI-based analysis of the surveyed articles. More specifically, we applied a hierarchical clustering approach to the collected documents. First, we manually created the dendrogram by collapsing the extracted articles into a smaller number of super-categories to reduce sparsity and harmonize language across the dataset, followed by grouping according to their shared super-categories. In parallel, we generated an AI-based dendrogram using hierarchical clustering. For each article, title (and when available, abstract) text was embedded using an AI-trained language model to obtain a vector representation of its semantic content. A more detailed method is presented in Appendix A.

**Figure 14:**
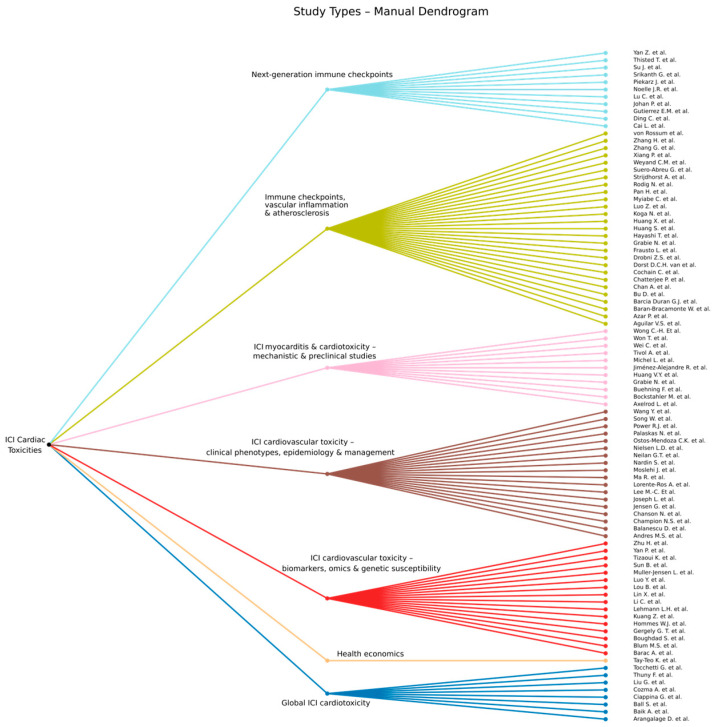
Manual dendrogram of study types in the ICI cardiac toxicities literature. The diagram shows hierarchical classification of all included articles, rooted at “ICI Cardiac Toxicities” and branching into seven super-categories: next-generation immune checkpoints; immune checkpoints, vascular inflammation and atherosclerosis; ICI myocarditis and cardiotoxicity—mechanistic and preclinical studies; ICI cardiovascular toxicity—clinical phenotypes, epidemiology and management; ICI cardiovascular toxicity—biomarkers, omics and genetic susceptibility; health economics; and global ICI cardiotoxicity. Each leaf node corresponds to a single publication (author label on the right), linked to its assigned category. Next-generation immune checkpoints: [24,25,26,27,28,29,30,31,32,33,34]. Immune checkpoints, vascular inflammation, and atherosclerosis: [9,10,11,12,13,21,23,35,36,37,38,39,40,41,42,43,44,45,46,47,48,49,50,51,52,53]. ICI myocarditis and cardiotoxicity—mechanistic and preclinical studies: [19,20,43,44,45,46,47,48,49,50,51,52,53,54,55,56]. ICI cardiovascular toxicity clinical phenotypes, epidemiology, and management: [2,3,4,17,57,58,59,60,61,62,63,64]. ICI cardiovascular toxicity biomarkers, omics & genetic susceptibility: [22,65,66,67,68,69,70,71,72,73,74,75,76,77,78,79]. Health economics: [1]. Global ICI cardiotoxicity: [6,7,14,15,16,80,81].

The rationale for the manual dendrogram helps to create a clinically and mechanistically meaningful grouping, ensuring that articles are clustered according to cardio-oncology concepts (phenotype, mechanism, vascular vs. myocardial focus, biomarker vs. trial design) rather than superficial textual similarity (Figure 14).

However, expert-driven schemes can be biased by prior assumptions. The AI-based hierarchical clustering provides a complementary, unsupervised view: articles are grouped solely based on their semantic proximity in embedding space, allowing emergent clusters to be identified and areas of disagreement with the manual taxonomy to be highlighted. Using both approaches in parallel strengthens the robustness of our organizational framework for ICI cardiovascular literature.

**Figure 15:**
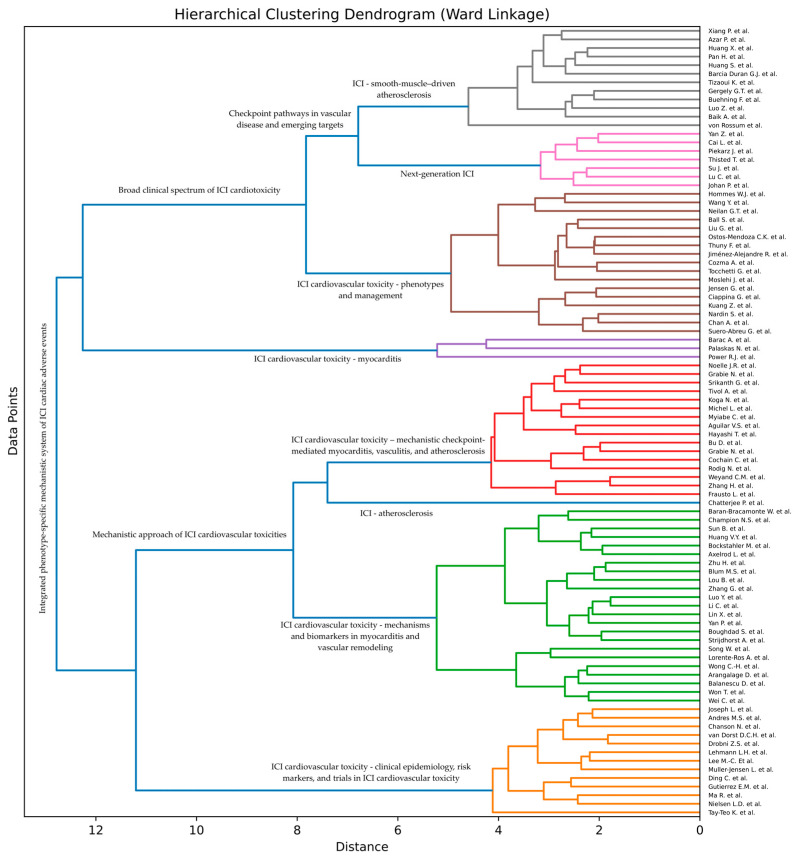
AI system dendrogram. *X*-axis (horizontal): the *X*-axis represents the distance or dissimilarity between different clusters of articles. As we move from 12 to 0 along the *X*-axis, the similarity between clusters increases, which means that the AI system searches for deep similarities between articles. We used a distance threshold of 13 to color the clusters. *Y*-axis (vertical): the *Y*-axis represents the data points (articles) in our dendrogram. Each data point is positioned vertically based on its cluster or subgroup. Clusters: articles that are close to each other on the dendrogram are more similar to each other, while articles that are farther apart are less similar. The clusters formed in the dendrogram indicate groups of articles with similar characteristics or attributes. Authors ordered from top to bottom: [1,2,3,4,6,7,8,9,10,11,12,13,14,15,16,17,19,20,21,22,23,24,25,26,27,28,29,30,31,32,33,34,35,36,37,38,39,40,41,43,44,45,46,47,48,49,50,51,52,53,54,55,56,57,58,59,60,61,62,63,64,65,66,67,68,69,70,71,72,73,74,75,76,77,78,79,80,81,83,84].

To generate an automatically constructed taxonomy of the surveyed studies, we employ a hierarchical clustering technique. Hierarchical clustering is an unsupervised machine learning method that can organize data points into a hierarchical structure (dendrogram) comprising multiple levels of clustering. At a certain level in the hierarchy, a data point can belong to a single cluster (group). At upper levels, data points are clustered in fewer coarse-level groups. At lower levels, coarse-level groups get divided into fine-grained groups. We apply a bottom-up (agglomerative) construction algorithm, starting with each data in a separate group. The algorithm iteratively merges clusters two by two, until all data points are gathered into one large group. At each iteration, two groups are selected for merging based on a linkage criterion. Researchers have explored multiple linkage criteria [82], each with its own benefits and drawbacks. In this study, we considered a range of popular alternative options, namely, single linkage, complete linkage, average linkage, centroid linkage and Ward linkage (Figure 15).

Figure 15 depicts the dendrogram generated through an unsupervised clustering pipeline that categorizes articles based on their similarity in BERT-derived embeddings. BERT, a comprehensive pre-trained language model, effectively captures broad semantic similarities between text samples. However, neither the embeddings nor the clustering process is specifically optimized for immune-checkpoint-inhibitor-related cardiovascular toxicities. Consequently, the algorithm may produce groupings that do not fully align with domain-relevant concepts. This limitation is not unique to our approach; it is inherent to unsupervised methods in general. Therefore, domain experts must review the dendrogram to ensure its meaningfulness. To address this, we manually examined the dendrogram (Figure 15) and cross-validated its structure against our expert-curated dendrogram (Figure 14).

Comparing the handmade dendrogram with the AI system, we see that the AI system grouped the studies well, validating our approach of studying cardiovascular toxicities from a mechanistic point of view, with both of these branches converging into an integrated phenotype-specific mechanistic system of ICI cardiac adverse events. However, while both recover the same high-level structure, they differ in how they partition the “border zone” articles. Studies that we manually placed in broad cardiotoxicity overviews or clinical phenotypes (Baik et al. [80], Ball et al. [81], Nardin et al. [57], Moslehi et al. [58], Muller-Jensen et al. [65]) are split by the AI system: some remain in a “clinical phenotypes and management” cluster, whereas others migrate toward the myocarditis or mechanistic/biomarker clusters because their abstracts emphasize specific pathways (PD-1/PD-L1, CXCR3–CXCL9/10, TCR clonality) or omics-driven risk stratification. Conversely, a number of articles that we grouped under “ICI—vascular inflammation and atherosclerosis” (Drobni et al. [9], Dorst et al. [12], Cochain et al. [35], Bu et al. [36], Barci Duran et al. [37], Zhang et al. [23], Azar et al. [38], Xiang et al. [39], Pan et al. [35]) are split by the AI between a proximal “ICI—atherosclerosis” node and a more upstream “smooth-muscle-driven atherosclerosis” node, reflecting differences in whether the abstract foregrounds SMC phenotypic switching and tumor-like clonal behavior, classical plaque biology, or ICI exposure per se. The AI system also sharpens the mechanistic branch. Articles on myocarditis genetics, immune systems, and detailed immunophenotyping (Tizaoui et al. [66] on PTPN22, Muller-Jensen et al. [65] and Lin et al. [67] on HLA, Yan et al. [24] and Zhu et al. [68] on TCR spectrum, Boughdad et al. [69] and Huang et al. [41] on cytokine/chemokine axes, Li et al. [70], Luo et al. [71] on ROCK2 and proteomics) are pulled together into a dense “mechanisms and biomarkers in myocarditis and vascular remodeling” cluster, rather than being dispersed across myocarditis and vascular subchapters as in the manual tree. At the same time, the AI isolates a relatively pure epidemiology, pharmacovigilance, and health economics cluster (Palaskas et al. [2], Andres et al. [5], Wang et al. [3], Nielsen et al. [4], Ma et al. [59], Joseph et al. [60], Tay-Teo et al. [1]), which we had distributed between “clinical phenotypes” and “broad cardiotoxicity” in the manual taxonomy. Finally, the next-generation checkpoint studies (Johan et al. [25], Lu et al. [26], Su et al. [27], Cai et al. [28], Srikanth et al. [29], Piekarz et al. [30], Gutierrez et al. [31], Yan et al. [24], Noelle et al. [32], Thisted et al. [33], Ding et al. [34]) form a compact “Next-generation ICI” subtree in both dendrograms.

Taken together, the AI-assisted clustering did not reveal new biological pathways independently of expert interpretation, but it did help refine the thematic organization of the literature. In particular, it recovered the major conceptual axes already recognized in the review—myocardial inflammation, vascular inflammation/atherosclerosis, biomarkers/genetics, and clinical epidemiology—while also separating finer semantic neighborhoods such as a myocarditis biomarker/genetics hub, a smooth-muscle-centered atherosclerosis cluster, and an epidemiology/health-systems block. These findings support the use of AI-assisted clustering as a literature organization tool within a broader review workflow.

## 7. Discussion

### 7.1. Cardiac Cellular Domain and Immune Checkpoint Expression

#### 7.1.1. Cardiomyocytes

Across phenotypes, our curated network intersects with a T cell–centric view of cardiac irAE. Our data show that CD8^+^ and CD4^+^ T lymphocytes, together with macrophages and CD3^+^ T cells, are the most frequently reported immune cells, while cardiomyocytes, endothelial cells and cardiac-resident macrophage subsets appear as the dominant structural components. This pattern fits well with the biopsy series of ICI myocarditis, where dense CD3^+^/CD8^+^ T-cell infiltrates, followed by CD68^+^ macrophages, are the defining histological feature, while the CD68/CD3 ratio is often increased [61]. Functionally, this cellular architecture is consistent with the cytokine milieu most often implicated in cardiac irAEs: IFN-γ is primarily produced by activated CD8^+^ cytotoxic T cells, Th1-polarized CD4^+^ T cells, and NK cells, whereas TNF-α is largely derived from activated monocytes/macrophages, acting as a feed-forward amplifier of endothelial activation and leukocyte recruitment. Upstream, IL-12 and the IL-12/23 axis are mainly produced by antigen-presenting cells, particularly dendritic cells and macrophages, reinforcing Th1 differentiation and IFN-γ production; IL-2 is secreted predominantly by activated CD4^+^ T cells and supports intramyocardial T-cell expansion once checkpoint restraint is removed. In parallel, IL-6 is produced early by myeloid cells such as monocytes, macrophages, and dendritic cells and can also be released by inflamed cardiac stromal/vascular compartments (endothelium and fibroblast-like cells), linking innate activation to downstream T-cell and acute-phase effector programs. Furthermore, the genes and molecules that bridge multiple phenotypes are overwhelmingly immune checkpoints and their effector circuits. PDCD1 (PD-1), CTLA4 and CD274 (PD-L1) dominate the gene network alongside LAG3 and TIM-3, while the molecule-level network highlights PD-1, PD-L1, CTLA-4 and their costimulatory molecules such as CD28, CD80 and CD86 as the most recurrent nodes. This mirrors experimental data showing that PD-1/PD-L1 and CTLA-4 are essential for restraining autoreactive T cells in the heart: PD-1 or combined PD-1/CTLA-4 deficiency in mice leads to spontaneous, often fatal myocarditis with CD4^+^/CD8^+^ infiltration, and genetic or pharmacologic disruption of PD-1/PD-L1 signaling lowers the threshold for autoimmune and inflammatory cardiomyopathy [42,54,83]. We capture extensive use of endothelial adhesion molecules (ICAM-1, VCAM-1, E-selectin) and NF-κB–linked cytokines in atherosclerosis and vasculitis, whereas myocarditis is uniquely enriched for cardiac structural antigens (α-myosin, troponin I, desmin) that may function as tissue-specific autoantigens. These patterns align with experimental work showing that PD-L1 is inducibly expressed on cardiac endothelial cells and cardiomyocytes in response to IFN-γ and TNF-α, where it acts as a local brake on CD8^+^ T-cell-mediated injury; loss or blockade of PD-L1 on these stromal cells predisposes to immune-mediated myocardial damage [43,44,45]. Molecules such as PD-1/PD-L1, CTLA-4, LAG-3 and their downstream cytokines (TNF-α, IFN-γ, IL-6, IL-12/23, IL-17) form a shared hub that connects myocarditis, vasculitis and atherosclerosis simultaneously, while conduction abnormalities and pericarditis are largely linked into this same hub rather than via unique signaling modules. An emerging clinical series in which dual PD-1/PD-L1 plus CTLA-4 blockade, which maximally perturbs this hub, is associated with the highest incidence and severity of cardiovascular irAEs across phenotypes [2,6]. Because immune checkpoints differ substantially in the maturity of cardiovascular evidence, the pathway-level interpretation should not treat all targets as equivalent. PD-1/PD-L1 has the strongest direct support from human ICI myocarditis studies, cardiac and vascular preclinical models, endothelial/cardiomyocyte PD-L1 biology, and atherosclerosis literature. CTLA-4 has strong immunological plausibility and clinical relevance, particularly in combination regimens, but less cardiac tissue-specific evidence than PD-1/PD-L1. In contrast, LAG-3, TIGIT, TIM-3, VISTA, and BTLA are mainly supported by oncology development data and general immunological rationale, with limited direct evidence linking them to specific cardiovascular phenotypes (Table 1).

#### 7.1.2. Vascular Smooth Muscle

Within our organ/tissue network, the arterial wall and atherosclerotic plaque emerged as dominant non-myocardial sites, and many of the molecules that connect these nodes to myocarditis and vasculitis are traditionally drivers of vascular smooth muscle cell (VSMC) activation and phenotypic switching (TNF-α, IFN-γ, IL-1β, IL-6, PD-1/PD-L1, adhesion molecules) [38,40,93]. Experimental and human plaque studies show that VSMCs undergo extensive phenotypic plasticity in atherosclerosis, down-regulating contractile markers and acquiring synthetic, inflammatory, osteogenic, or macrophage-like phenotypes under pro-atherogenic stimuli [46]. Single-cell and lineage-tracing research now supports a model in which clonal expansion of VSMCs drives much plaque growth, leading some authors to characterize atherosclerosis as a “smooth-muscle-cell-driven, tumor-like disease” [47,48]. In mice, KLF4-dependent phenotypic modulation is a key regulatory switch that enables VSMCs to downshift contractile programs and enter alternative inflammatory/synthetic states; SMC-specific Klf4 deletion reduces SMC-derived macrophage-like/mesenchymal states and is associated with smaller lesions and features of improved plaque stability. In humans, intimal SMCs carry a substantial fraction of the plaque cholesterol burden and can generate cells that are misclassified as macrophages by canonical markers, highlighting why CD68 positivity is not lineage-definitive in plaque tissue [94]. Additionally, modulated VSMCs do not collapse into one terminal fate; instead, VSMC-derived macrophage-like cells can display transient or branching fates, with subsets adopting fibroblast/pericyte-like features later in the disease [95].

Moreover, immune checkpoint pathways are increasingly recognized as critical regulators of VSMC behavior. In atherosclerosis-prone mice, PD-1 deficiency amplifies T-cell activation and increases lesional T-cell accumulation, tipping the balance toward inflammatory plaque remodeling and accelerated atherogenesis [35]. This heightened T-cell state is relevant to VSMC biology because inflammatory cytokine pressure and contact-dependent immune interactions are established triggers of VSMC remodeling. Complementarily, agonistic stimulation of PD-1 promotes atherosclerotic lesion development by modulating adaptive immune responses, consistent with a protective role of coinhibitory signaling in plaque immunity [84]. Downstream of heightened effector activation, T cells can directly drive plaque vulnerability: CD8^+^ cytotoxic T lymphocytes promote features of vulnerable plaques via perforin and granzyme-dependent apoptosis of vascular cells including smooth muscle cells, with consequent necrotic core formation and amplification of inflammatory signaling [96]. Human studies show that CD4^+^ T cells can establish stable contacts with VSMCs and induce VSMC apoptosis, a mechanism that can directly translate excessive T-cell effector activity into mural-cell loss and plaque destabilization; type I interferon–primed CD4^+^ T cells can further acquire cytotoxicity toward VSMCs through TRAIL-dependent pathways [97,98]. Moreover, macrophage-mediated death pathways can overlap on the same endpoint: monocyte/macrophage FasL signaling can induce VSMC apoptosis in atherosclerotic lesions, providing an innate effector route to VSMC depletion and cap weakening [99]. Importantly, fibroblast activation protein (FAP) signal has also been evaluated in vascular disease contexts as a distinct plaque-associated pattern relative to canonical contractile markers and other inflammation/ECM-turnover markers, and it has been explored as a potential in vivo readout of activated/modulated smooth muscle biology [100]. In vitro, IFN-γ induces PD-L1 expression on vascular smooth muscle cells, and blockade of PD-1/PD-L1 signaling augments T-cell–driven VSMC proliferation, linking the checkpoint directly to neointimal growth [49,50]. In murine models, genetic or pharmacologic impairment of the PD-1 axis enhances T-cell activation, increases plaque inflammation and accelerates atherosclerosis, whereas normal PD-1 signaling restrains pro-atherogenic T-helper responses [35,51]. Recent single-cell research in human coronary atherosclerosis demonstrates PD-1 expression not only in naive CD4^+^ T cells but also in myofibroblasts and smooth-muscle-like cells within the plaque, emphasizing that PD-1/PD-L1 signaling is involved in the stromal as well as immune pathways of the atherosclerotic wall [36,37].

### 7.2. Immune Checkpoint Pathways: Mechanistic Position and Cardiac Implications

In concordance with the literature, our network emphasizes that CTLA-4 is primarily connected to lymphoid and systemic immune nodes, whereas PD-1/PD-L1 is expressed on myocardial, endothelial, and atherosclerotic tissue, consistent with the dominant role of PD-1/PD-L1 signaling in maintaining peripheral cardiac immune tolerance [101,102]. Preclinical and translational studies show that PD-L1 is constitutively or injury-inducibly expressed on cardiomyocytes and cardiac endothelium, and that genetic deletion or pharmacologic disruption of PD-1/PD-L1 signaling amplifies T-cell activation, augments myocardial inflammation and worsens outcomes after cardiac injury [21,44,52]. Human biopsy and autopsy series of ICI myocarditis show dense infiltrates of cytotoxic CD8^+^ T cells and CD68^+^ macrophages with PD-L1-positive cardiomyocytes, supporting a model in which checkpoint blockade removes a local inhibitory shield at the myocardial interface and allows for unrestrained cytotoxic and myeloid effector activity [58,62]. Arrhythmic manifestations, including high-grade atrioventricular block and malignant ventricular arrhythmias, are common in ICI myocarditis and reflect the direct involvement of the conduction system, as documented by electrocardiographic cohorts and case series that highlight conduction disease as a presenting feature and major determinant of early mortality [44,83]. However, few data are present in the current literature regarding the exact pathophysiology of these conduction abnormalities, even regarding preclinical models or clinical datasets.

### 7.3. Predicting Toxicity Patterns for Current and Future ICIs

Our framework may suggest that predicting cardiovascular toxicity patterns for current and future ICIs will require layering molecular biomarkers and genetic risk markers onto specific myocardial, conduction-system, and vascular modules, rather than applying uniform surveillance algorithms to all patients and regimens [72,81,103]. Cardiomuscular damage markers are the most promising candidates: troponin elevation is present in ~90–95% of ICI myocarditis cases, and time-resolved curves of cTnI/cTnT and CK correlate with major adverse cardiac events, supporting baseline and serial troponin as a quantitative readout of injury within the myocarditis or NILVD phenotypes highlighted in our network [2,73,74]. Beyond necrosis markers, circulating cytokine and chemokine molecules are emerging as dynamic indicators of checkpoint status: small cohorts of ICI myocarditis show consistent elevations of IL-6 and IL-10, with diagnostic studies suggesting IL-6 and CXCL13 as relatively sensitive markers and CXCL9/CXCL10 as more specific for ICI myocarditis versus other inflammatory states, pointing directly to the CXCR3–CXCL9/10 axis that occupies central T cell–endothelial interfaces in our network [15,55,56]. Omics approaches are now nominating more specific protein biomarkers that align with nodes in our network: single-cell RNA-seq and bulk integration in patients with ICI myocarditis identify the S100A protein family as a potential serum marker reflecting activated innate immune clusters; plasma proteomic studies distinguish ICI myocarditis from acute myocardial infarction based on inflammation- and complement-related proteins; and a dedicated study of Rho-kinase 2 (ROCK2) reports promising diagnostic performance for ICI-associated myocarditis, all of which link CD68^+^ myeloid, PD-1^+^ effector T-cell, and endothelial modules in our network map [70,71,72]. At the genetic level, first reports of HLA associations and dedicated genetic programs support a future gene diagnosis layer for personalizing ICI choice and monitoring intensity: the extended HLA-A01:01-B08:01-C07:01 haplotype has been linked to early-onset severe myocarditis/myositis under ICIs; studies with myositis and myocarditis report enrichment of HLA-DQB103:03, HLA-C01:02, and HLA-B52:01; humanized DQ8 models develop fulminant myocarditis or myositis under dual checkpoint blockade; and a clinical trial (“Genetic Determinants of Myocarditis Induced by ICIs,” NCT06734689) is now systematically approaching HLA class I/II associations [63,65,67,104]. Although no specific non-HLA susceptibility SNPs have yet been validated for ICI myocarditis, extensive data linking PTPN22, CTLA-4, PDCD1, and ICOS polymorphisms to systemic autoimmunity provide a rationale for future polygenic risk scores anchored in the dendritic cell–T-cell priming and PD-1^+^ effector hubs that our network identifies as candidate upstream regulatory nodes of myocarditis, vasculitis, and accelerated atherosclerosis phenotypes [66,75]. Complementary studies come from TCR and single-cell multi-omics: in ICI myocarditis, TCR-β sequencing demonstrates oligoclonal expansion of CD8^+^ T cells with shared clones between tumor and myocardium, while peripheral blood studies show expansion of effector/Temra CD8^+^ subsets expressing heart-tropic chemokine receptors; in preclinical Ctla4^+/−^Pdcd1^−/−^ models, integrated scRNA-seq, TCR-seq, and spatial transcriptomics map these clones to PD-1^+^ effector–cardiomyocyte and effector–endothelium synapses, matching the dominant edges in our myocarditis and vasculitis phenotypes [41,69,77,78]. Single-cell banks of human ICI myocarditis cardiac tissue and peripheral blood mononuclear cells (PBMCs), together with vascular scRNA-seq datasets, further refine organ-specific vulnerability predictions by delineating fibroblast, endothelial, VSMC, and immune clusters that overexpress PD-L1, CXCL9/10, CXCL2, and S100A family members—precisely the chemokine and stromal nodes that our network places at the intersection of myocarditis, vasculitis and accelerated atherosclerosis [53,78,79,105]. Table 2 provides a mapping of each biomarker to the most frequently associated phenotype, the predominant evidence level, and the proposed clinical utility (screening, early detection, phenotype, risk stratification and monitoring response).

Our study has several limitations. First, it was designed as a narrative review rather than a systematic review, and, therefore, the included literature does not represent the full clinical literature on ICI-related cardiovascular toxicities; we did not prospectively register a protocol, conduct a formal risk-of-bias assessment, or pool clinical effect estimates. Second, the framework is inherently literature driven and thus inherits the biases of the field, particularly the overrepresentation of myocarditis and the relative underdevelopment of mechanistic data for conduction disease, NILVD, microvascular injury, and several vasculitic phenotypes. The proportional maps reflect literature representation rather than causal hierarchy. Frequently represented entities such as PD-1, PD-L1, CTLA-4, TNF-α, and IL-6 may be prominent because they are biologically important, but also because they are heavily studied, easily measured, and repeatedly discussed in the ICI literature. Conversely, newer or phenotype-specific mediators may appear less often because they remain underexplored. Although record-level deduplication prevents repeated mentions within a single article from inflating the counts, it cannot correct for field-level publication bias, research-popularity bias, or unequal assay availability. Thus, proportional representation should be interpreted as literature prominence and mechanistic plausibility, not causal importance. Third, the curated network integrates heterogeneous evidence from preclinical models, translational studies, case-based observations, and cohort-level clinical data; as a result, the proposed links should be interpreted as a synthesis of plausibility and convergent evidence rather than as uniformly causal, directionally validated pathways. Fourth, the AI-assisted component was restricted to semantic clustering of the included literature and should therefore be understood as an organizational complement to expert review rather than an autonomous source of biological inference. Finally, the proposed biomarkers and risk markers remain at different levels of maturity and require prospective validation with standardized phenotype definitions, longitudinal biosampling, tissue-based profiling, and external replication before they can support clinical-grade risk stratification.

Several areas now require dedicated validation to strengthen the proposed mechanistic model. Prospective cardio-oncology cohorts with standardized definitions of myocarditis, NILVD, conduction disease, vasculitis, and accelerated atherosclerosis are needed to confirm whether these phenotypes truly segregate into distinct biomarker and pathway modules. Longitudinal sampling studies should test whether candidate markers such as troponin, IL-6, CXCL9/CXCL10/CXCL13, S100A-family proteins, ROCK2, soluble PD-L1, HLA-linked susceptibility signals, and TCR-clonality measures track onset, severity, and recovery in a phenotype-specific manner. In parallel, tissue-level, single-cell, and spatial studies are needed to validate the proposed cardiomyocyte, endothelial, and VSMC circuits and to determine whether the links identified here are causal, therapeutically actionable, or both.

To summarize, these data and our framework may point toward a future precision cardio-oncology workflow in which (i) pre-treatment HLA analysis and autoimmune-gene panels related to ICIs may point to a specific myocardial or vascular damage, (ii) baseline and serial troponin plus promising cytokines or chemokines such as IL-6, CXCL9/10, CXCL13, myeloid-derived proteins (S100A), and pathway markers may provide an individualized checkpoint injury liability, and (iii) TCR/single-cell profiling may be used in high-risk regimens (CTLA-4 + PD-1, PD-1 + LAG-3) to detect expansion of pathogenic clones. This will allow clinicians to anticipate whether a given patient is more likely to develop a myocarditis phenotype versus predominantly vascular toxicities such as vasculitis or ICI-accelerated atherosclerosis under specific checkpoint combinations.

### 7.4. Strength and Consistency of Evidence Across Study Types

The evidence supporting ICI-related cardiovascular toxicity is heterogeneous, and not all mechanistic conclusions have the same level of support. Some conclusions are relatively consistent across study types. The association between ICIs and myocarditis is supported by case series, pharmacovigilance analyses, observational cohorts, pathology studies, and meta-analyses, although reported incidence varies according to case definition, surveillance intensity, treatment regimen, and patient population [2,6,58,61]. Human biopsy and autopsy studies consistently describe a T-cell- and macrophage-rich inflammatory pattern, with CD3^+^/CD8^+^ T-cell infiltration and CD68^+^ macrophages as recurrent tissue findings [109,110]. In parallel, preclinical and translational studies support a biologically plausible role for PD-1/PD-L1 signaling in maintaining myocardial immune tolerance, particularly through endothelial and cardiomyocyte PD-L1–mediated restraint of CD8^+^ T-cell injury [21,43,44,45]. These convergent data support the conclusion that ICI myocarditis is a clinically relevant immune-mediated toxicity in which PD-1/PD-L1 and CTLA-4 pathway disruption can contribute to loss of cardiac immune tolerance.

Other conclusions are supported by convergent but incomplete evidence. The link between ICI therapy and accelerated atherosclerosis is supported by observational event-based studies, serial imaging data, and preclinical atherosclerosis models [35,36,37,80]. However, the relative contribution of direct checkpoint disruption, systemic inflammation, baseline cardiovascular risk, cancer-related inflammation, and concomitant therapies remains incompletely defined [10,11,111]. Similarly, endothelial activation, leukocyte trafficking, and vascular smooth muscle cell remodeling are biologically plausible mechanisms connecting checkpoint inhibition to vascular inflammation, but the specific role of these pathways in ICI-exposed patients remains less mature than the evidence for myocarditis [10]. Therefore, vascular conclusions should be interpreted as mechanistically plausible and clinically relevant, but not yet fully validated at the same level as myocarditis.

Several areas remain preliminary or hypothesis-generating. NILVD is increasingly recognized as a clinical phenotype, but its mechanistic separation from myocarditis is not yet firmly established. Proposed mechanisms, including cytokine-mediated myocardial depression, microvascular dysfunction, stress-cardiomyopathy-like injury, and unmasking of pre-existing ventricular vulnerability, remain incompletely validated [7,112]. Conduction abnormalities are clinically important, especially in the context of ICI myocarditis, but dedicated mechanistic studies of conduction tissue are sparse [64,113]. Likewise, VSMC phenotypic switching is well established in general atherosclerosis biology, but its specific role in ICI-associated plaque progression is still largely inferential [114,115,116].

The evidence base for biomarkers is similarly uneven. High-sensitivity cardiac troponin is the most clinically mature marker and is already used in the assessment and monitoring of suspected ICI myocarditis [73,74]. By contrast, IL-6, IL-10, CXCL9/CXCL10, and CXCL13 are supported by smaller clinical or translational studies and should be considered candidate adjunctive inflammatory markers rather than established diagnostic tools [22,55,56,69]. S100A-family proteins, ROCK2, soluble PD-L1, endothelial activation markers, HLA associations, non-HLA autoimmune-linked polymorphisms, and TCR-clonality measures remain translational or hypothesis-generating. These markers may help refine future mechanistic subtyping, but they are not yet validated for routine risk prediction, surveillance, or treatment selection [65,66,67,68,70,75,78,107,108].

Finally, emerging checkpoint pathways differ substantially in evidence maturity. PD-1/PD-L1 has the strongest direct cardiovascular evidence, supported by human myocarditis studies, preclinical cardiac and vascular models, and endothelial/cardiomyocyte PD-L1 biology [21,43,44,45,58,80]. CTLA-4 has strong immunological plausibility and clinical relevance, especially in combination regimens, but it also has less cardiac tissue-specific evidence than PD-1/PD-L1 [6,85,86,87]. In contrast, LAG-3, TIGIT, TIM-3, VISTA, and BTLA are supported mainly by oncology development data and general immunological rationale, with limited direct evidence linking these pathways to defined cardiovascular phenotypes [28,31,32,33,34,92]. Accordingly, these emerging checkpoints should be interpreted as future cardio-oncology considerations rather than established mechanistic drivers of ICI-related cardiovascular toxicity.

## 8. Materials and Methods

### 8.1. Study Design and Literature Search

This study takes the form of a narrative review. A comprehensive literature search was conducted in MEDLINE (via PubMed), Embase, Scopus, and Web of Science to capture preclinical and clinical data on cardiovascular toxicity arising from modulation of co-inhibitory immune checkpoints. We used free-text keywords for immune checkpoint pathways (“immune checkpoint inhibitor”, “programmed cell death 1”, “PD-1”, “programmed death ligand 1”, “PD-L1”, “cytotoxic T-lymphocyte-associated protein 4”, “CTLA-4”, “lymphocyte activation gene 3”, “LAG-3”, “TIGIT”, “TIM-3”, and cardiovascular outcome terms (“myocarditis”, “pericarditis”, “vasculitis”, “arrhythmia”, “conduction disorder”, “atrioventricular block”, “non-inflammatory left ventricular dysfunction”, “cardiomyopathy”, “heart failure”, “atherosclerosis”, “coronary artery disease”).

### 8.2. Eligibility Criteria

Studies were included if they reported at least one of the following: (i) preclinical or translational evidence linking checkpoint pathways to myocardial, vascular, endothelial, pericardial, or conduction-system injury; (ii) clinical cardiovascular immune-related adverse events occurring during immune checkpoint inhibitor therapy; (iii) molecular, cellular, genetic, biomarker, imaging, or tissue-level data relevant to ICI-associated cardiovascular toxicity; or (iv) review-level synthesis useful for contextualization or reference mining.

Studies were excluded if they: (i) addressed ICIs without cardiovascular outcomes; (ii) described cardiovascular disease without immune checkpoint biology or ICI exposure; (iii) reported non-oncological immune checkpoint biology without relevance to cancer therapy; (iv) lacked extractable mechanistic, phenotypic, or clinical information; (v) were duplicate publications, conference abstracts without sufficient data, editorials without mechanistic content, or non-accessible full texts; or (vi) focused exclusively on non-cardiovascular immune-related adverse events.

### 8.3. Evidence Weighting and Role in Synthesis

Because this review aimed to construct a mechanistic framework rather than estimate pooled clinical effect sizes, evidence types were not statistically weighted or formally graded. Instead, different study designs were interpreted according to their evidentiary role in the mechanistic synthesis. Preclinical and translational studies, including in vivo models, ex vivo systems, in vitro experiments, tissue profiling, single-cell studies, proteomics, and genetic analyses, were prioritized for pathway construction because they provide direct or near-direct mechanistic evidence. Clinical cohorts, registries, pharmacovigilance studies, and clinical trials were used primarily to define phenotype spectrum, temporal patterns, clinical relevance, and biomarker associations. Case reports and case series were used to capture rare phenotypes, phenotype boundaries, and mechanistic hypotheses, but they were not treated as sufficient evidence for causal pathway assignment unless supported by experimental, tissue-level, or convergent clinical data. Review articles were used for contextual framing, terminology harmonization, and citation mining; when primary studies were available, reviews were not counted as independent primary evidence for a mechanistic link.

### 8.4. Data Extraction and Entity Harmonization

Data from eligible records were manually extracted into a structured spreadsheet with predefined fields, including study characteristics, checkpoint pathway, drug or target, cardiovascular phenotype, genes, molecules, cell populations, tissues, proposed pathophysiology, and whether the record contributed to the systems-biology map. Gene and protein names were harmonized to HGNC-approved symbols when possible. Immunological markers were mapped to standardized cell-type or molecule categories. Synonyms were consolidated to reduce duplication, and semicolon-separated entries were used for multi-entity fields.

### 8.5. Record-Level Proportional Mapping of Mechanistic Entities

Record-level proportional mapping was used to summarize how often standardized entities appeared as curated mechanistic elements across the included literature. These percentages represent the proportion of included records in which a gene, molecule, cell type, tissue compartment, or phenotype was identified as mechanistically relevant after study-level deduplication. They should not be interpreted as measures of causal importance, biological centrality, effect size, clinical incidence, or pathogenic necessity. Highly represented entities may reflect true biological relevance, but they may also reflect research intensity, publication trends, assay availability, and the focus of the field on selected checkpoint and cytokine pathways. Therefore, record-level deduplication was used to prevent a single article from contributing repeated mentions of the same entity, but this strategy cannot correct for broader literature-level biases, including overrepresentation of well-studied pathways such as PD-1/PD-L1, CTLA-4, TNF-α, and IL-6.

### 8.6. Phenotypic Overlap Analyses

To identify shared and phenotype-restricted mechanisms, curated gene, molecule, and cell sets were compared across major cardiovascular phenotypes, including myocarditis, atherosclerosis, vasculitis, conduction abnormalities, and NILVD. Venn-based overlap analyses were used to distinguish common immune-injury cores from phenotype-specific mediators.

### 8.7. KEGG Pathway Enrichment Analysis

Pathway over-representation analysis was performed using the Kyoto Encyclopedia of Genes and Genomes (KEGG) as the reference. For the global analysis, we first constructed a non-redundant list of genes mapped to HGNC-approved symbols, preventing us from artificially inflating the weight of extensively studied targets and ensuring compatibility with gene-centric pathway databases. Non-gene entities and ambiguous tokens were excluded from KEGG enrichment and were interpreted solely at the qualitative network level. This gene set was tested for enrichment of KEGG pathways using a hypergeometric over-representation framework with the human genome as background. For per-phenotype analyses, separate gene lists were generated for myocarditis, pericarditis, vasculitis, conduction abnormalities, atherosclerosis, and NILVD, and each list was independently interrogated against KEGG. For each pathway, we recorded the KEGG term identifier, term name, intersection size (number of query genes in the pathway), query size, and effective background size, together with the associated *p*-value. Where relevant, multiple-testing correction was applied using the false discovery rate, and results are reported as –log_10_(p) in the figures. Dot plots summarize the most significantly enriched pathways, with dot position representing pathway identity, dot size proportional to intersection size, and dot color proportional to –log_10_(p). More data can be found at Supplementary File S2. All network constructions and visualizations were performed in Python 3.13.2 using networkx and matplotlib.

### 8.8. Integrated Phenotype–Molecule/Gene Network

A bipartite phenotype–molecule/gene network was constructed to integrate associations between cardiovascular phenotypes and curated mediators. Phenotype nodes represented myocarditis, pericarditis, vasculitis, conduction abnormalities, atherosclerosis, and NILVD. Molecular or gene nodes were connected to phenotypes when they were curated from the literature as mechanistically relevant. Node importance was defined as the number of distinct phenotypes connected to each mediator. Functional classes, including checkpoint molecules, checkpoint ligands, cytokines, chemokines, adhesion molecules, complement, immunoglobulins, T-cell markers, macrophage markers, metabolic/mitochondrial proteins, intracellular signaling mediators, and cardiac structural antigens, were assigned to molecular nodes when possible.

### 8.9. AI-Assisted Semantic Clustering

AI-assisted semantic clustering was used as an exploratory organizational tool for the included literature. Article titles and available abstracts were embedded using a language-model-based representation and clustered using hierarchical agglomerative clustering. Alternative linkage criteria were evaluated, and Ward linkage was selected based on interpretability of the resulting hierarchy. The AI-derived dendrogram was compared with an expert-curated taxonomy.

## 9. Conclusions

Our framework highlights several areas where validation and further studies need to be developed to strengthen the mechanistic model of the immune-related cardiovascular events. Current evidence suggests that myocarditis, NILVD, conduction disease, vasculitis, and accelerated atherosclerosis may share a checkpoint-driven inflammatory scaffold involving T-cell activation, myeloid amplification, cytokine signaling, and endothelial recruitment. At the same time, the available literature suggests phenotype-specific tissue contexts and mediators, particularly at the level of cardiomyocytes, cardiac endothelium, vascular smooth muscle cells, and atherosclerotic plaque. These conclusions are best interpreted as a mechanistic synthesis of heterogeneous preclinical, translational, and clinical evidence rather than as evidence of validated causal pathways for each phenotype.

Several areas require future validation. Prospective cardio-oncology cohorts with standardized phenotype definitions are needed to distinguish myocarditis, NILVD, conduction disease, vasculitis, and accelerated atherosclerosis more reliably. Longitudinal biosampling should test whether candidate biomarkers such as troponin, IL-6, CXCL9/CXCL10/CXCL13, S100A-family proteins, ROCK2, soluble PD-L1, HLA-linked susceptibility signals, and TCR-based markers track onset, severity, recovery, and recurrence in a phenotype-specific manner. Tissue-level, single-cell, and spatial studies are also needed to determine whether the pathway connections proposed here are causal, therapeutically actionable, or primarily associative. Therefore, risk-adapted monitoring, AI-driven multi-omics prediction, and phenotype-specific prevention strategies should currently be regarded as research directions rather than established clinical tools. If validated prospectively, these approaches may eventually support more individualized cardiovascular surveillance during ICI therapy. At present, however, clinical practice should continue to rely on established cardio-oncology assessment, symptom evaluation, electrocardiography, cardiac biomarkers such as troponin, cardiac imaging when indicated, and multidisciplinary management for suspected ICI-related cardiovascular toxicity.

## Figures and Tables

**Table 1:** Evidence of the maturity of established and emerging immune checkpoint pathways in cardiovascular toxicity.

Checkpoint Pathway	Type of Supporting Evidence	Cardiovascular Phenotypes Implicated	Current Evidence Maturity	Major Knowledge Gaps
PD-1/PD-L1 [4,9,43,45,80]	Human ICI myocarditis cohorts; preclinical knockout and blockade models; endothelial/cardiomyocyte PD-L1 studies; atherosclerosis models; translational biomarker and single-cell studies	Myocarditis; conduction abnormalities; NILVD; accelerated atherosclerosis; vasculitis/endothelial inflammation	Most mature direct cardiovascular evidence	Need prospective validation of phenotype-specific biomarkers; unclear mechanisms distinguishing myocarditis from NILVD and conduction disease; limited causal validation in human vascular phenotypes
CTLA-4 [6,85,86,87]	Clinical association with higher myocarditis risk in combination regimens; preclinical immune-tolerance models; evidence of enhanced T-cell priming and loss of immune restraint	Myocarditis; myocarditis–myositis overlap; severe systemic irAEs; possible conduction abnormalities when myocarditis is present	Moderate direct clinical evidence; strong immunological plausibility	Difficult to separate CTLA-4-specific effects from combined PD-1/CTLA-4 blockade; limited cardiac tissue-specific mechanistic data; unclear contribution to vascular phenotypes
LAG-3 [88,89,90]	Clinical exposure through approved LAG-3/PD-1 combination therapy; immunological studies of T-cell exhaustion and effector regulation; limited cardio-oncology-specific mechanistic data	Potential myocarditis or systemic irAEs, mostly in the setting of combination checkpoint blockade	Limited direct cardiovascular evidence	Cardiovascular phenotype spectrum remains poorly defined; limited tissue-level data; unclear whether LAG-3 blockade independently increases risk or modifies PD-1-related toxicity
TIGIT [28,91]	Oncology trials of TIGIT blockade, usually combined with PD-1/PD-L1 inhibitors; immunological rationale involving T-cell and NK-cell activation; minimal direct cardiovascular data	No well-defined cardiovascular phenotype; theoretical risk of inflammatory irAEs under combination therapy	Exploratory/indirect evidence	Lack of dedicated cardio-oncology safety analyses; no validated mechanistic cardiovascular model; unclear endothelial, myocardial, or plaque-level effects
TIM-3 [24,28,31]	Early oncology trials and preclinical immunology studies; role in T-cell exhaustion, myeloid regulation, and inflammatory signaling; limited cardiovascular toxicity reporting	No established ICI-related cardiovascular phenotype; possible relevance to inflammatory and myeloid-driven toxicity remains theoretical	Exploratory/indirect evidence	Direct cardiovascular toxicity data are sparse; unclear whether TIM-3 modulation affects myocarditis, vascular inflammation, or atherosclerosis under ICI therapy
VISTA [32,33]	Preclinical and early clinical immuno-oncology studies; VISTA expression on myeloid cells; theoretical relevance to inflammatory control	No established cardiovascular irAE phenotype	Exploratory/preclinical evidence	Unknown cardiovascular safety profile; limited human clinical exposure; unclear role in myocardial macrophage activation, vascular inflammation, or plaque biology
BTLA/HVEM [34,92]	Early clinical development in oncology; immunological evidence for T-cell inhibitory signaling; limited direct cardiovascular data	No established cardiovascular irAE phenotype	Exploratory/indirect evidence	No defined cardio-oncology phenotype; absence of dedicated mechanistic cardiovascular studies; unclear effect of BTLA modulation in combination regimens

**Table 2:** Proposed biomarkers for immune-checkpoint-inhibitor-associated cardiovascular toxicities.

Evidence Level	Biomarker	Phenotype	Clinical Utility
Already used in clinical monitoring	High-sensitivity cardiac troponin (hs-cTnI/hs-cTnT) [74]	Myocarditis	Early detection, monitoring response/relapse
Preclinical/translational mechanistic mediators	TNF-alfa, IFN-gamma [106]	Myocarditis	Monitoring
Preliminary clinical evidence	IL-6 (serum/plasma) [22,69]	Myocarditis	Early detection, response monitoring
Preliminary clinical evidence	IL-10 (serum/plasma) [22]	Myocarditis	Response monitoring
Preliminary clinical/translational evidence	CXCL13 (serum/plasma) [55]	Myocarditis	Complementary marker
Preliminary clinical/translational evidence	CXCL9/10 (serum/plasma) [56]	Myocarditis	Rule-in support for ICI myocarditis vs. NILVD
Translational exploration stage	S100A protein family [78,107]	Myocarditis	Future risk stratification
Translational/early clinical exploration	ROCK2 (Rho-kinase 2) [70]	Myocarditis	Future risk stratification
Future risk-stratification candidate	HLA-A01:01–B08:01–C07:01 [65]	Myocarditis	Predispozition, risk stratification
Preclinical susceptibility model	DQ8 model susceptibility [67]	Fulminant myocarditis	Support for HLA-linked risk
Translational/preclinical exploration	Soluble PD-L1 [108]	Immune activation marker	Risk stratification
Translational/preclinical exploration	Endothelial activation markers [45]	Vasculitis; accelerated atherosclerosis	Monitoring inflammation
Future mechanistic subtyping candidate	Oligoclonal CD8^+^ [68]	Myocarditis	Blood-based diagnosis, monitoring, relapse stratification
Future risk-stratification candidate	Non-HLA autoimmune-linked polymorphisms (PTPN22, CTLA-4, PDCD1) [66,75]	Risk layer across myocarditis/vasculitis/accelerated atherosclerosis	Risk assessment

## Data Availability

Data supporting the reported results can be found at https://doi.org/10.6084/m9.figshare.30936515.

## Supplementary Materials

The following supporting information can be downloaded at: https://www.mdpi.com/article/10.3390/ijms27104378/s1.

## Abbreviations

The following abbreviations are used in this manuscript:

AI	Artificial intelligence
ICIs	Immune checkpoint inhibitors
CTLA-4	Cytotoxic T-lymphocyte-associated protein-4
PD-1	Programmed cell death-1
PD-L1	Programmed death-ligand 1
LAG-3	Lymphocyte-activation gene 3
TIGIT	T-cell immunoreceptor with Ig and ITIM domains
TIM-3	T-cell immunoglobulin and mucin domain-containing protein 3
VISTA	V-domain Ig suppressor of T cell activation
BTLA	B- and T-lymphocyte attenuator
KEGG	Kyoto Encyclopedia of Genes and Genomes
irAE	Immune-related adverse event
irAEs	Immune-related adverse reactions
NILVD	Non-inflammatory left ventricular dysfunction
VSMC	Vascular smooth muscle cell
PBMCs	Peripheral blood mononuclear cells
TCR	T-cell receptor
Th1	T helper type 1 cells
Th17	T helper 17 cells
Tregs	Regulatory T cells
iNOS	Inducible nitric oxide synthase
ICAM-1	Intercellular adhesion molecule-1
VCAM-1	Vascular cell adhesion molecule-1
MYH6	Myosin heavy chain 6
Ldlr	Low-density lipoprotein receptor
APOE	Apolipoprotein E
RAG1	Recombination activating gene 1
RAG2	Recombination activating gene 2
FOXP3	Forkhead box P3
miR-34a	microRNA-34a
TNF-α	Tumor necrosis factor-alpha
IFN-γ	Interferon-gamma
IL-1β	Interleukin-1 beta
IL-6	Interleukin-6
IL-12	Interleukin-12

## Appendix A

Let $C_{i}$ and $C_{j}$ be two clusters that are candidates for the merging operation, at a given iteration of the agglomerative clustering algorithm. Single linkage selects the clusters that minimize the distance between the closest two data points belonging to different clusters. Formally, the single linkage criterion is defined as follows:

$$\Delta \left(C_{i},C_{j}\right)=\underset{x\in C_{i},y\in C_{j}}{min}\left\{\Delta \left(x,y\right)\right\},$$

where x and y are data points from the dataset, and Δ is the Euclidean distance. Similarly, complete linkage aims to minimize the distance between the farthest two data points, i.e.,

$$\Delta \left(C_{i},C_{j}\right)=\underset{x\in C_{i},y\in C_{j}}{max}\{\Delta (x,y)\}.$$

Average linkage computes the distances between all pairs of data points taken from different clusters and aims to minimize the average of these distances:

$$\Delta \left(C_{i},C_{j}\right)=\frac{1}{\left|C_{i}\right|\cdot \left|C_{j}\right|}\sum_{x\in C_{i}}\sum_{y\in C_{j}}\Delta (x,y).$$

Instead of computing distance between all pairs, centroid linkage first computes the centroid of each cluster, then merges the clusters with the minimum distance between their centroids:

$$\Delta \left(C_{i},C_{j}\right)=\Delta \left(\frac{\sum_{x\in C_{i}}x}{\left|C_{i}\right|},\frac{\sum_{y\in C_{j}}y}{\left|C_{j}\right|}\right)=\Delta (\mu_{C_{i}},\mu_{C_{j}}),$$

where $\left|C\right|$ is the number of data points in a cluster C, and $\mu_{C}$ is the centroid of cluster C. Finally, Ward linkage choses to merge the clusters with the smallest increase in variance, effectively minimizing the following objective:

$$\Delta \left(C_{i},C_{j}\right)=\frac{1}{\left|C_{i}\cup C_{j}\right|}\sum_{x\in C_{i}\cup C_{j}}\Delta (x,\mu_{C_{i}\cup C_{j}})-\frac{1}{\left|C_{i}\right|}\sum_{y\in C_{i}}\Delta \left(y,\mu_{C_{i}}\right)-\frac{1}{\left|C_{j}\right|}\sum_{z\in C_{j}}\Delta \left(z,\mu_{C_{j}}\right)$$

$$Var\left(C_{i}\cup C_{j}\right)-Var\left(C_{i}\right)-Var\left(C_{j}\right),$$

where ∪ represents the merging operation.

Upon evaluating the various linkage criteria, we observed distinct behaviors, e.g., single linkage tends to produce long, chain-like hierarchies, while complete linkage tends to produce compact clusters. Among all linkage criteria, we found the criterion that provides the best results on our dataset of research articles is Ward linkage, as it handles clusters with various densities better than the other criteria.

So far, we did not elucidate how research articles are transformed into data points that undergo clustering. We hereby consider two alternative text representations, a conventional method based on term frequency-inverse document frequence (TF-IDF), and a deep learning method based on Bidirectional Encoder Representations from Transformers (BERT) [106].

TF-IDF is a high-dimensional sparse vector, where each element indicates the importance of a certain word (term) from a pre-established vocabulary. More specifically, the importance is computed as:

$$TF-IDF\left(t,d\right)=TF\left(t,d\right)\cdot IDF\left(t\right),$$

where t is a term from the vocabulary, d is a document, and TF and IDF are two functions defined below. Term Frequency (TF) measures how often a word appears in a document:

$$TF\left(t,d\right)=\frac{\mathrm{count}\;\mathrm{of}\;\mathrm{term}\;t\;\mathrm{in}\;\mathrm{document}\;d}{\mathrm{total}\;\mathrm{terms}\;\mathrm{in}\;d},$$

while Inverse Document Frequency (IDF) downweights common words across the document collection:

$$IDF\left(t\right)=log\frac{N}{\mathrm{number}\;\mathrm{of}\;\mathrm{documents}\;\mathrm{containing}\;t},$$

where *N* is the total number of documents in the dataset, i.e., the number of surveyed papers.

BERT is a deep neural language model based on transformers, which benefits from pre-training on large corpora. The input text is split into tokens and processed by a transformer model based on bidirectional self-attention, which outputs the same number of tokens. A special token *[CLS]* is typically used for discriminative downstream tasks. We therefore represent each text document via the corresponding *[CLS]* token returned by the BERT model. We employ the BERT-base-uncased model, which uses tokens of 768 dimensions.

While TF-IDF has a low computational cost, it has several disadvantages that might affect the clustering process. First, it ignores word order and context, e.g., collapsing phrases such as “cancer likes sugar” and “sugar likes cancer” into the same representation. Second, it does not handle synonyms and polysemy well, e.g., failing to assign the same representation to “cancer treatment” and “tumor treatment”. In contrast, BERT can represent semantic relations into the embedding space, being able to handle synonymy, antonymy, hypernymy and other such relations. We tested both TF-IDF- and BERT-based representations on the gathered collection of research articles. We found the clustering based on BERT embeddings to enable an easier interpretation of the resulting hierarchy. Hence, in this survey, we only present and discuss the dendrogram obtained with BERT embeddings.
