# Supplementary figures and images for: Immune-Checkpoint-Inhibitor-Related Cardiovascular Toxicities in Cancer: A Mechanistic Review of Molecular Pathways with AI-Assisted Literature Clustering
Source: Int J Mol Sci. 2026 May 14;27(10):4378. doi: 10.3390/ijms27104378 (PMC13206904; doi:10.3390/ijms27104378)

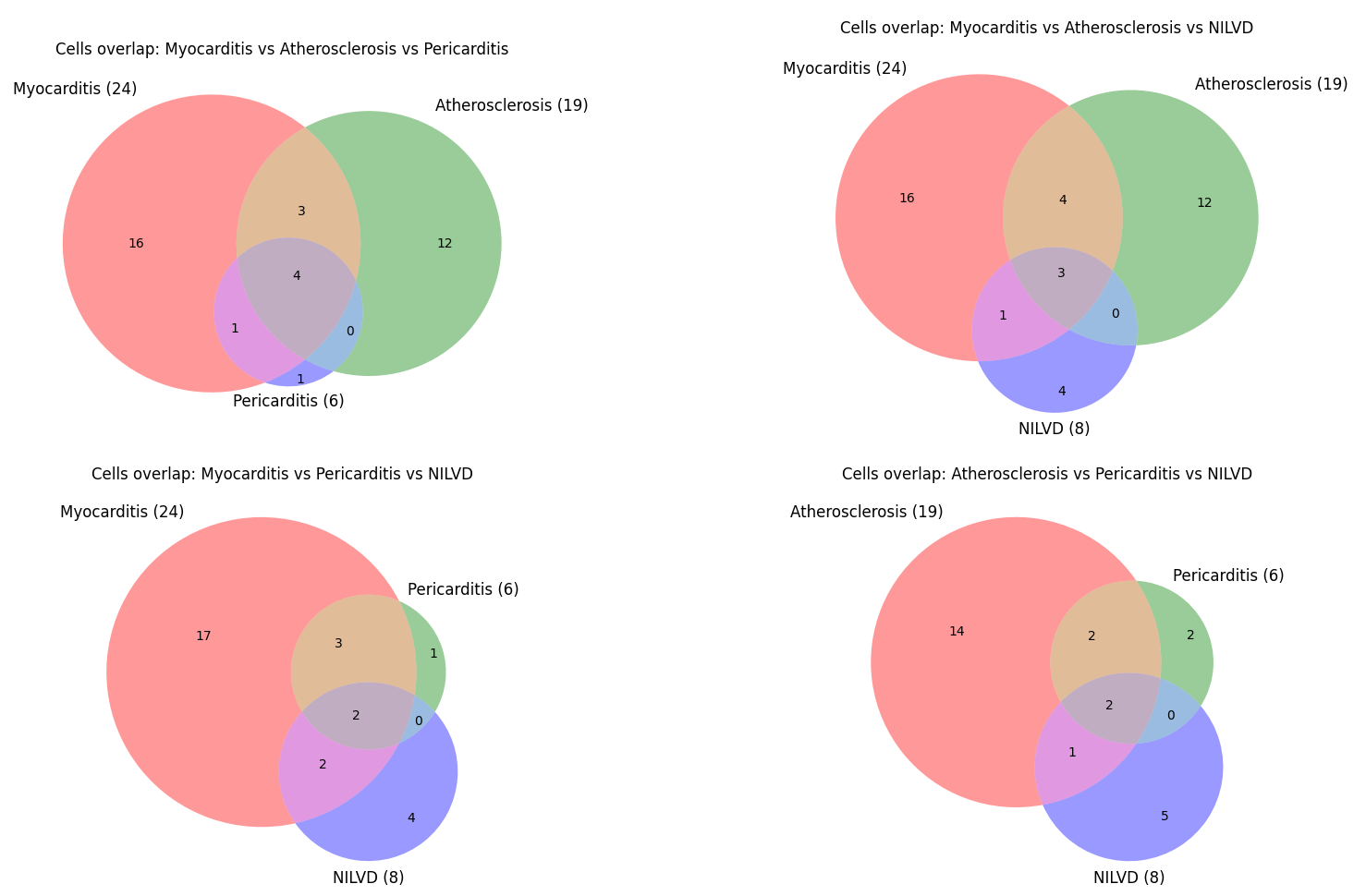

Supplement: Supplementary file 1 [file ijms-27-04378-s001.zip › Supplementary File S1/Venn_Cells_V1.jpeg]

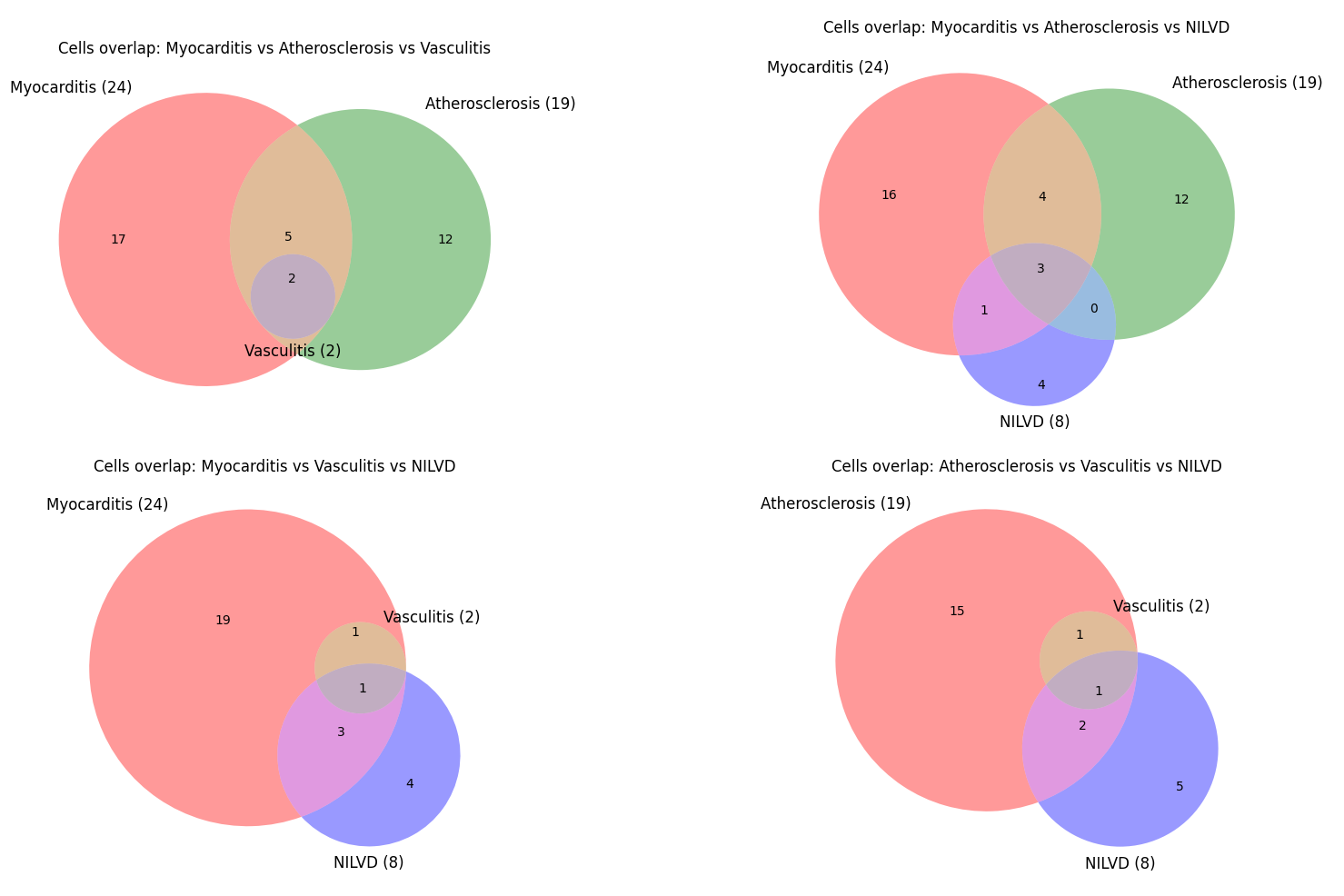

Supplement: Supplementary file 1 [file ijms-27-04378-s001.zip › Supplementary File S1/Venn_Cells_V2.png]

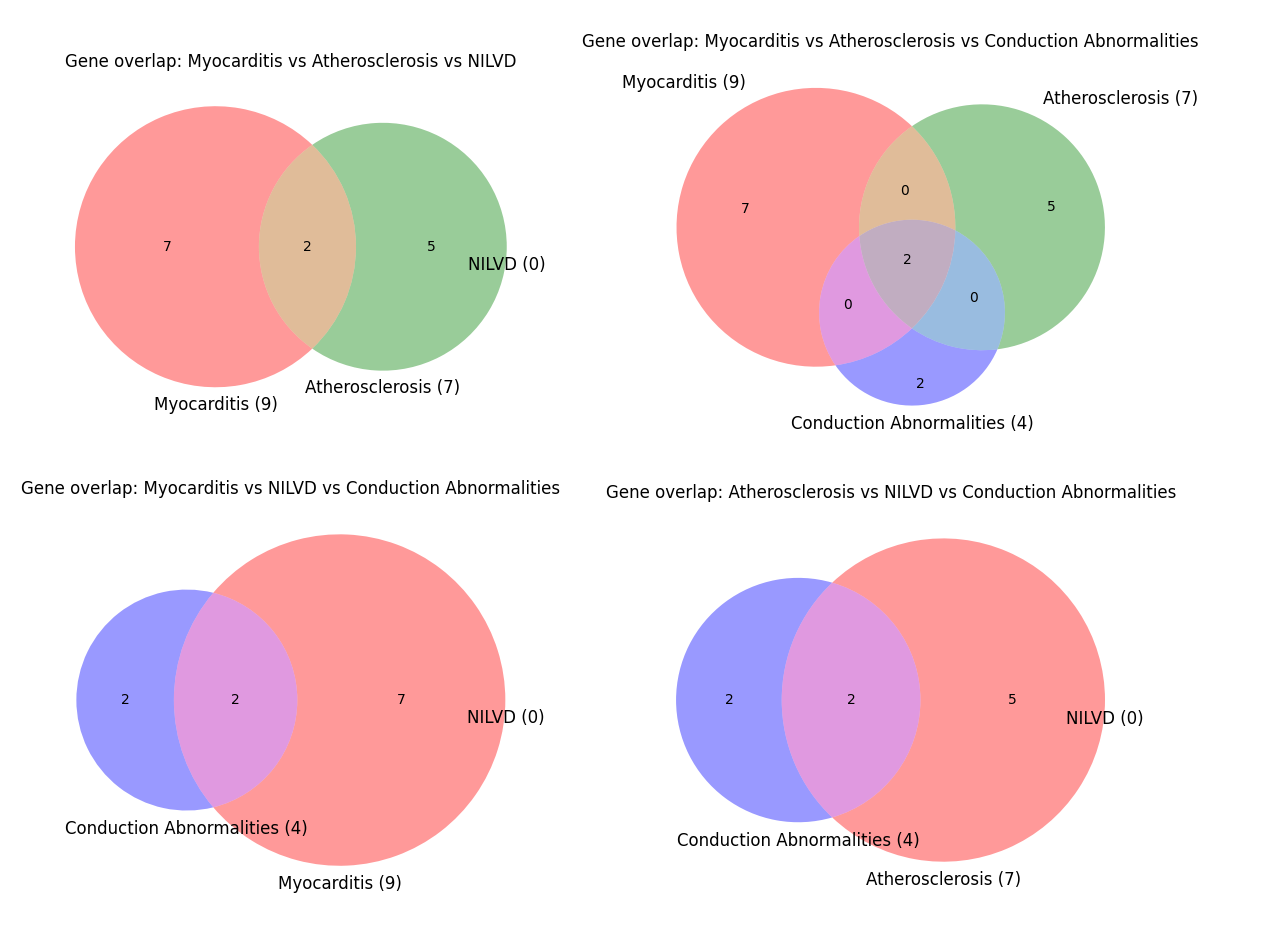

Supplement: Supplementary file 1 [file ijms-27-04378-s001.zip › Supplementary File S1/Venn_Diagram_3sets_V1_genes.png]

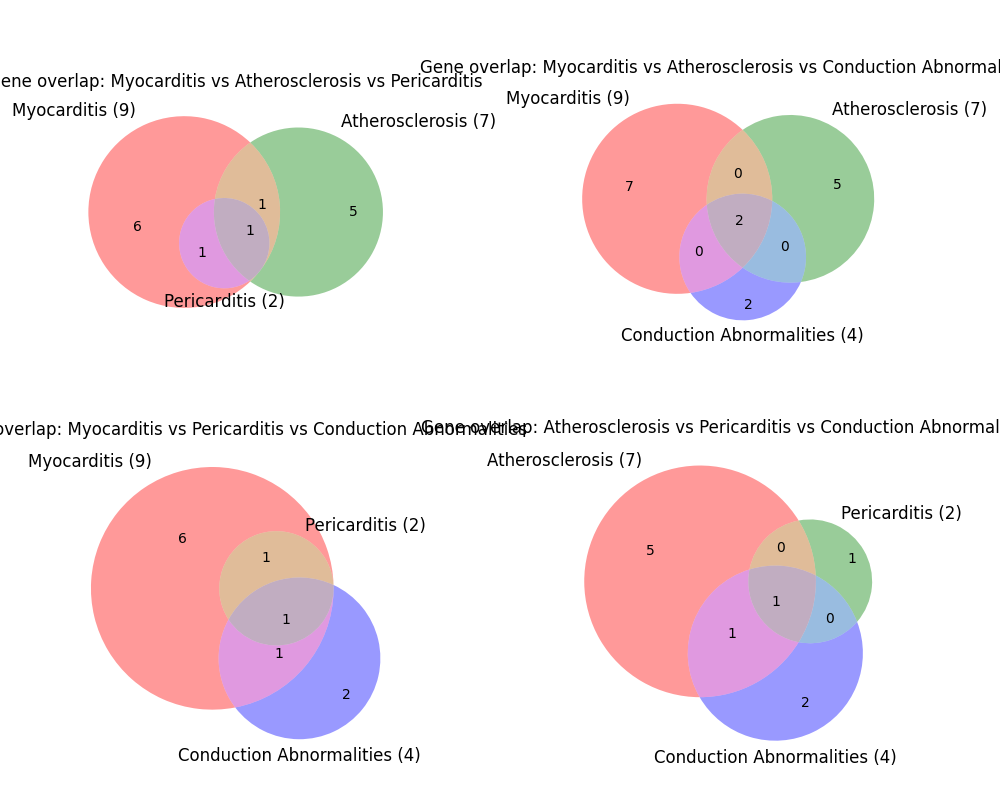

Supplement: Supplementary file 1 [file ijms-27-04378-s001.zip › Supplementary File S1/Venn_Diagram_3sets_V2_genes.png]

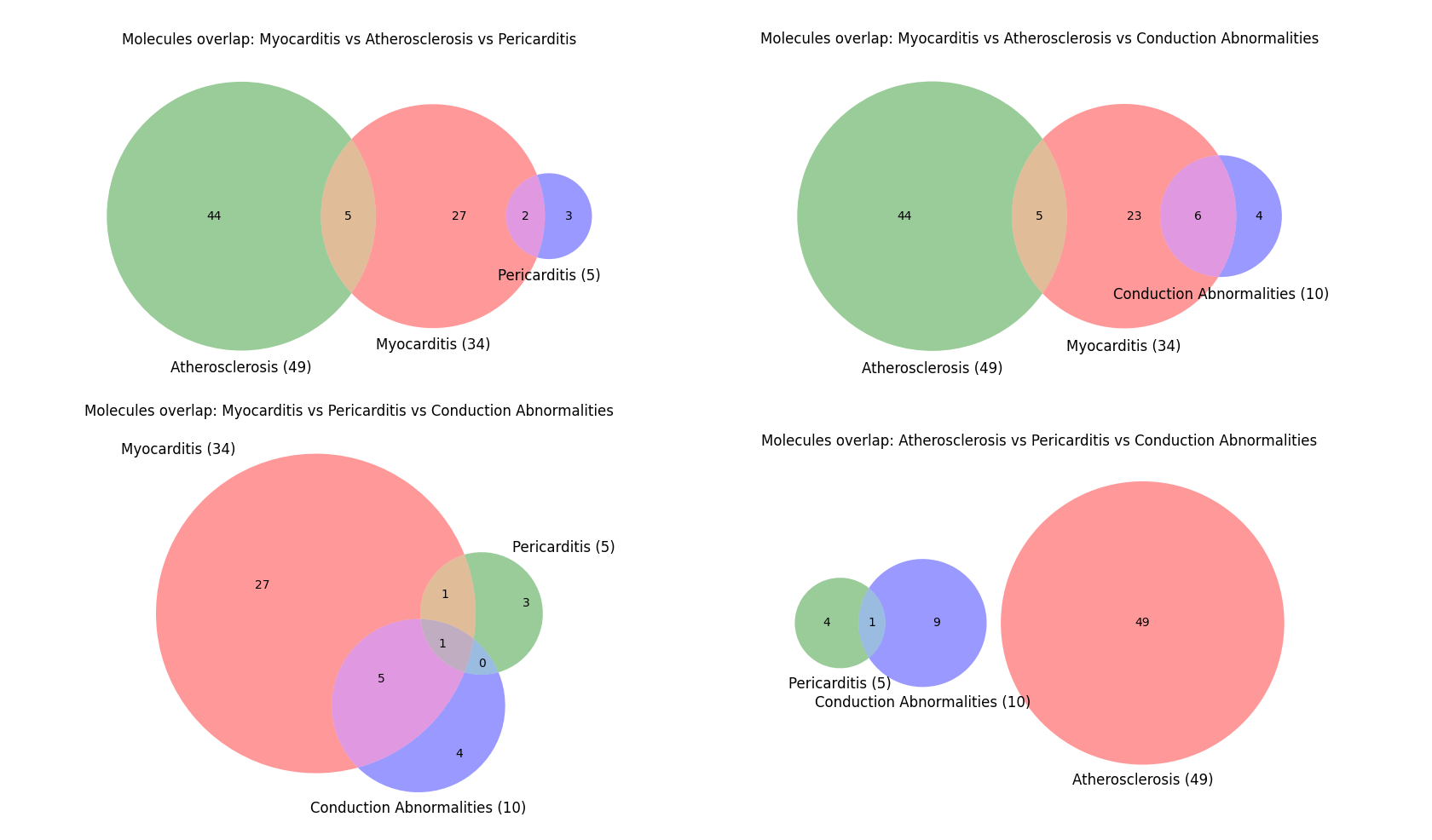

Supplement: Supplementary file 1 [file ijms-27-04378-s001.zip › Supplementary File S1/Venn_Molecules_V1.png]

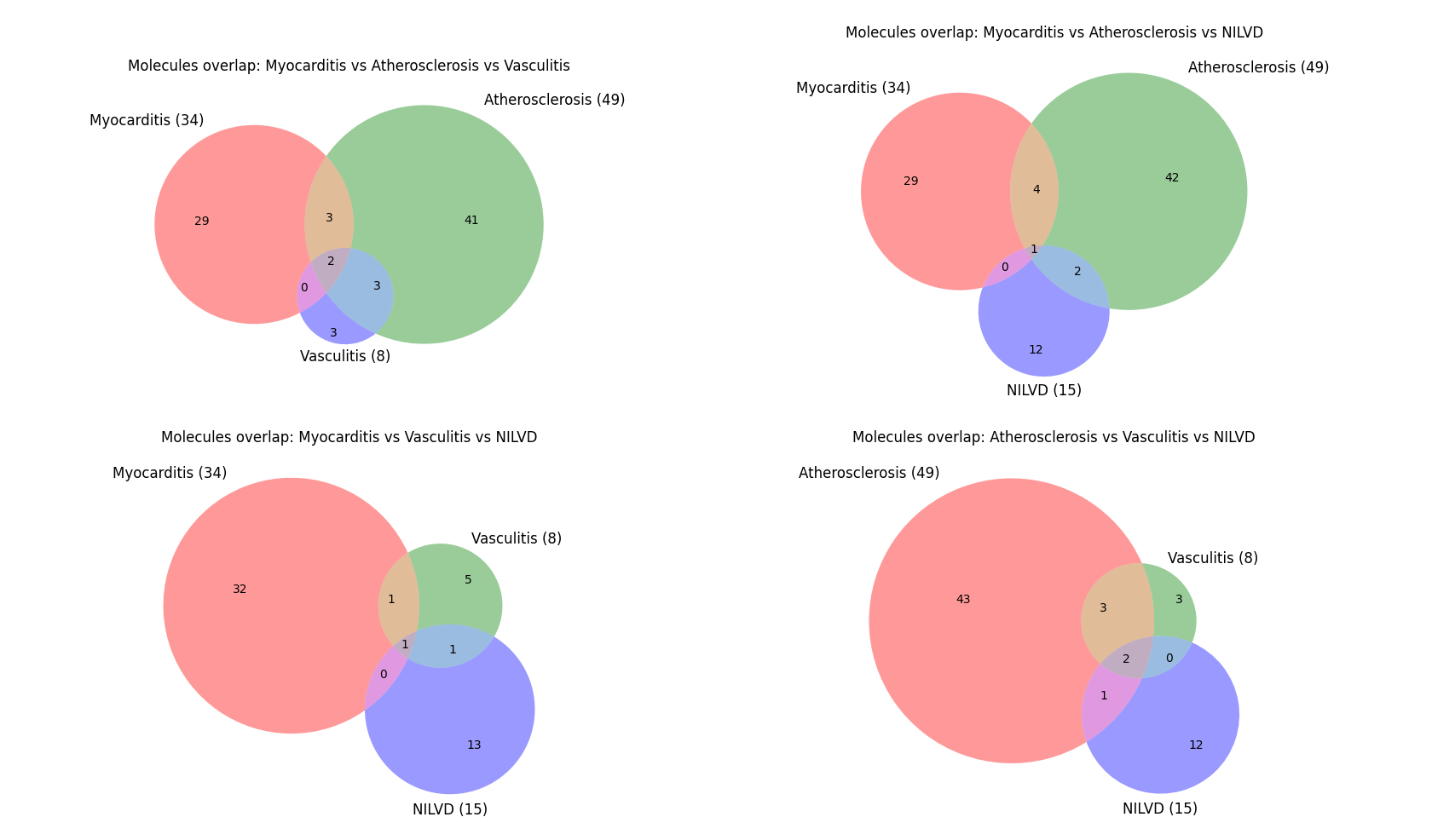

Supplement: Supplementary file 1 [file ijms-27-04378-s001.zip › Supplementary File S1/Venn_Molecules_V2.png]

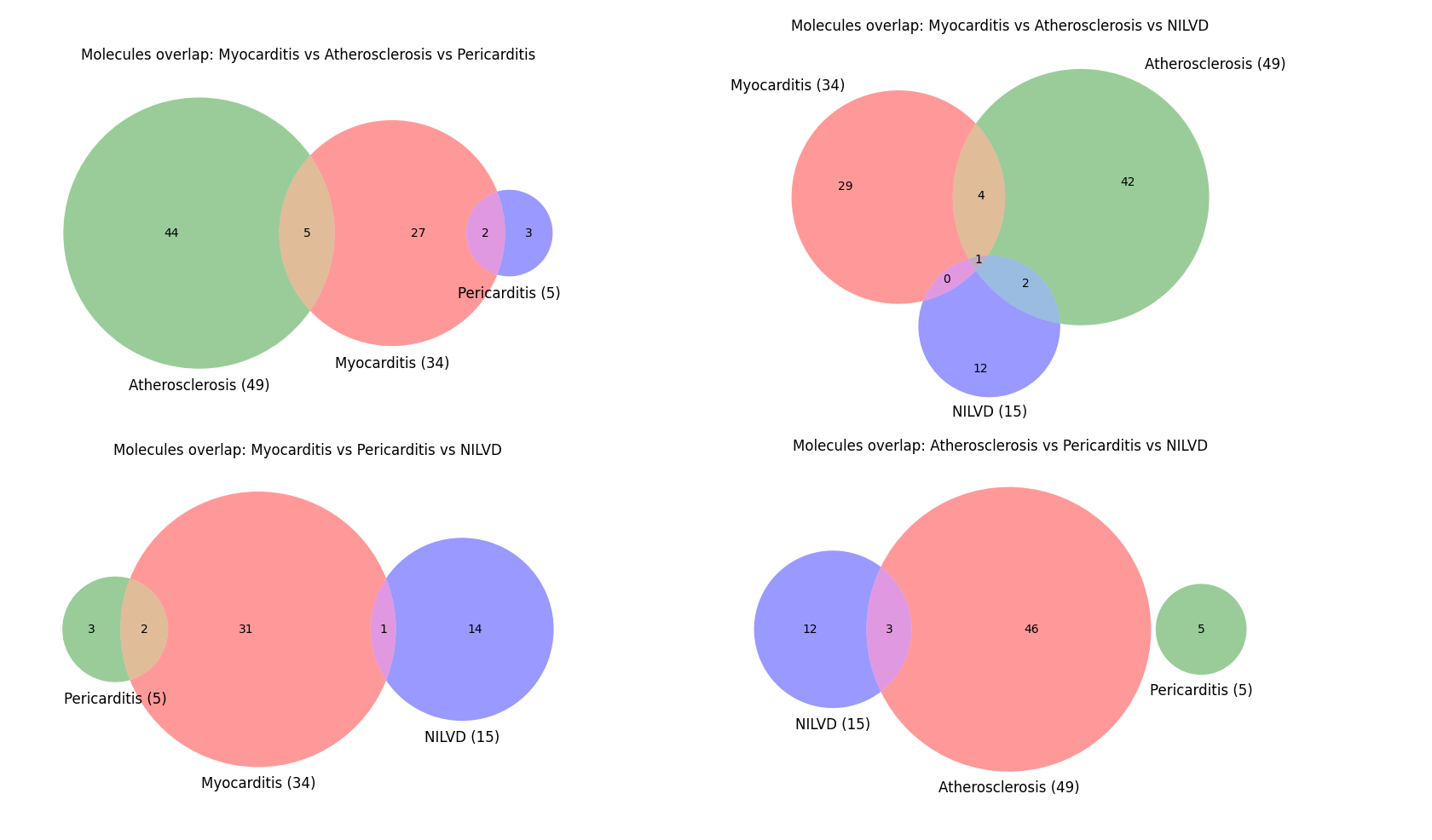

Supplement: Supplementary file 1 [file ijms-27-04378-s001.zip › Supplementary File S1/Venn_Molecules_V3.png]

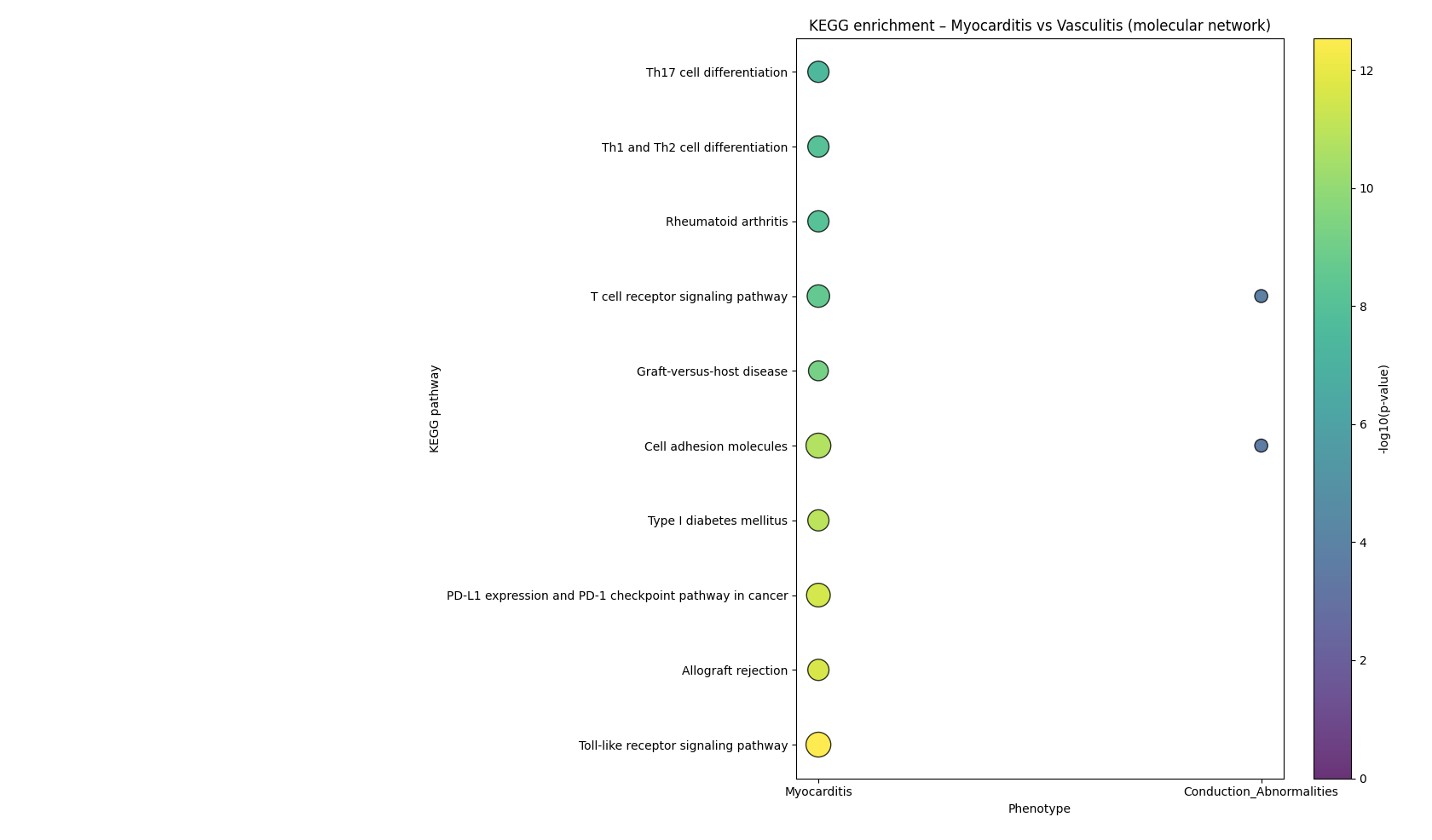

Supplement: Supplementary file 1 [file ijms-27-04378-s001.zip › Supplementary File S2/KEGG_Phenotype_DotPlot_V2.png]

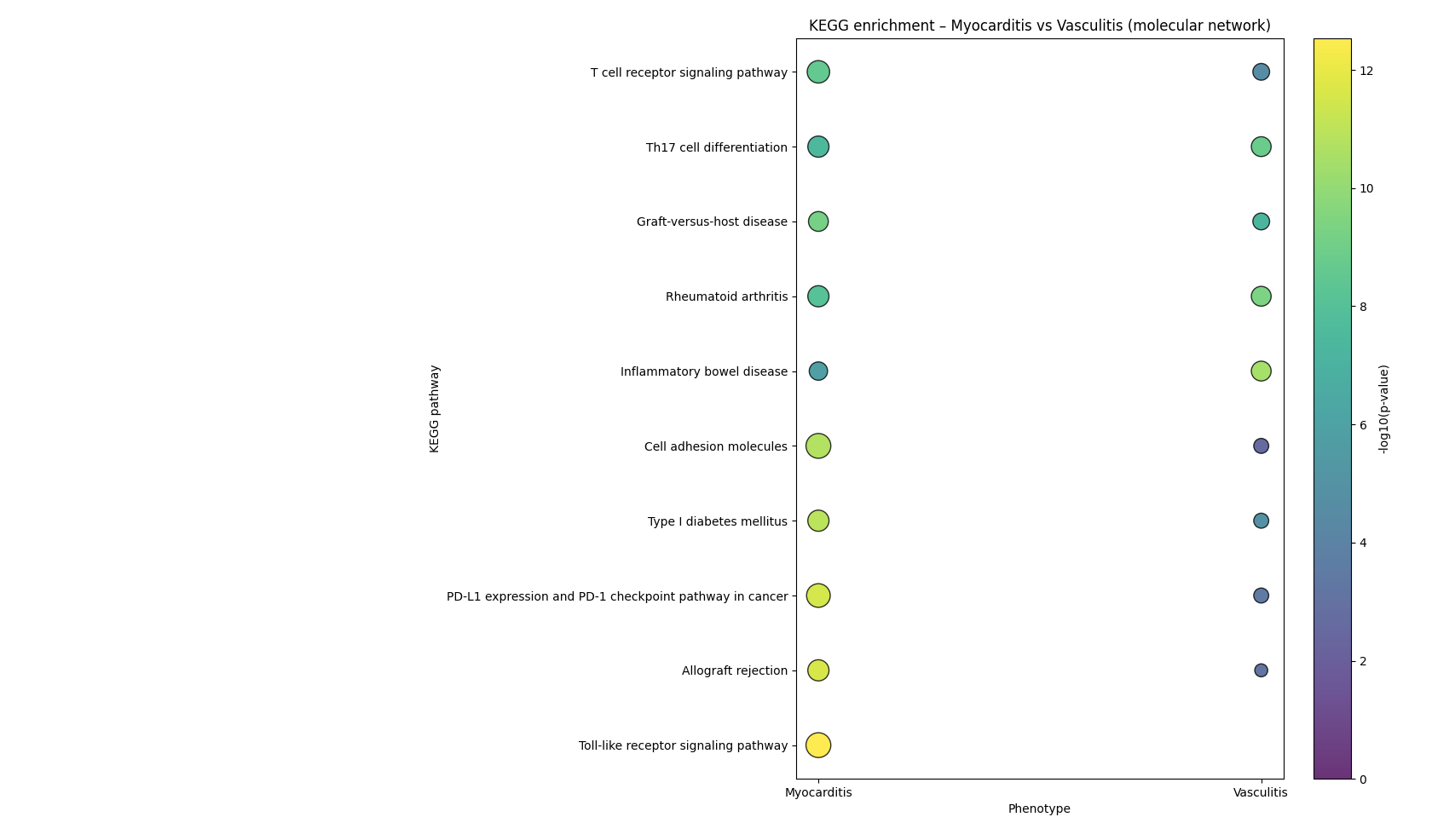

Supplement: Supplementary file 1 [file ijms-27-04378-s001.zip › Supplementary File S2/KEGG_Phenotype_DotPlot_V3.png]

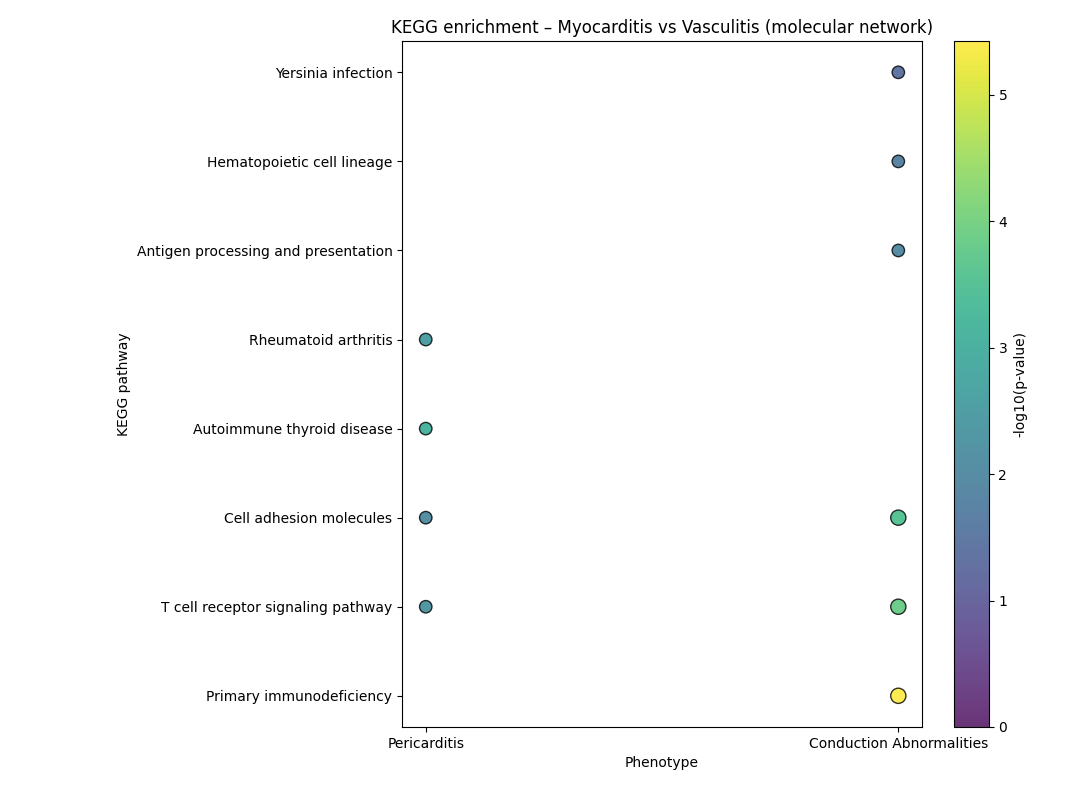

Supplement: Supplementary file 1 [file ijms-27-04378-s001.zip › Supplementary File S2/KEGG_Phenotype_DotPlot_V5.png]
